# Need for Cognition Predicts Academic Interest Development but Not the Other Way Around: A Longitudinal Study of Secondary School Students

**DOI:** 10.1111/cdev.14262

**Published:** 2025-06-04

**Authors:** Julia Matthes, Vsevolod Scherrer, Franzis Preckel

**Affiliations:** ^1^ Department of Psychology Trier University Trier Germany

**Keywords:** academic interest, investment traits, motivational development, need for cognition, secondary school

## Abstract

Need for cognition (NFC) reflects the tendency to enjoy and engage in cognitive challenges. This study examines the relations between NFC and academic interest among 922 German secondary school students (academic track) assessed four times in Grades 5–7 (initial age *M* = 10.63, SD = 0.55; 41% female; 90% first language German) in mathematics, German, and English. Data were collected between 2008 and 2012 and were analyzed using autoregressive cross‐lagged panel models. In all domains, NFC positively predicted subsequent academic interest (β = 0.03 to β = 0.17) but interest did not positively predict subsequent NFC. Findings were comparable after controlling for students' achievement, gender, socioeconomic status, and class type. They suggest that NFC is a potential facilitator of the development of academic interest in school.

Need for cognition (NFC) and academic interest are important constructs for understanding the academic development of children and adolescents in school. They are positively related to academic achievement (for meta‐analyses, see Liu and Nesbit 2023; Schiefele et al. 1992) and impact how students engage in class and enjoy their time in school even in the absence of explicit extrinsic rewards (Lavrijsen et al. 2021; Murayama 2022; Renninger and Hidi 2016). NFC refers to “stable individual differences in people's tendency to engage in and enjoy effortful cognitive activity” (Cacioppo et al. 1996). Interest describes a state of engagement in a situation as well as the persistent motivation to revisit specific content (Krapp 2002; Renninger 2000); academic interest refers to interest in content related to the academic field. According to the Four‐Phase Model of Interest Development by Hidi and Renninger (2006), academic interest develops from situationally triggered interest to stable individual interest. This development is influenced by person‐related and environmental variables. NFC may be one of these person‐related variables, but it has rarely been investigated in the context of interest development. Person‐related variables that support the development of interests include positive affect, sustained attention, persistence, or content knowledge (Renninger and Hidi 2016). Cross‐sectionally, NFC is positively related to these variables and also to academic interest (e.g., Petty et al. 2009; Preckel 2014). In addition, according to the Intelligence‐as‐Process, Personality, Interest, and Intelligence‐as‐Knowledge (PPIK) theory of intellectual development (Ackerman 1996), NFC supports the development of individual academic interest by enhancing an individual's cognitive engagement and persistence. Academic interest, on the other hand, contributes to continued exploration, learning, and understanding of specific content (Renninger and Hidi 2016), which may create opportunities for engaging in and enjoying effortful cognitive activities, thereby supporting the development of NFC. Thus, it is plausible that students' NFC contributes to their academic interest development and vice versa. Although NFC and academic interest have been linked in several cross‐sectional studies, longitudinal evidence of their relation is scarce. The present study aims to address this gap by examining the reciprocal relations between NFC and academic interest. Knowledge about reciprocal relations could enhance our understanding of the development of central variables related to engagement and learning in schools. This knowledge may enable the evaluation of interventions aimed at supporting learners' academic development and the design of new interventions to equip students with the best possible tools to further their learning.

## 1 Need for Cognition, Academic Interest and Their Development

### 1.1 Need for Cognition

Individuals with high NFC “engage in and enjoy effortful cognitive activity” (Cacioppo et al. 1996, 197). They enjoy to actively search for new information and reflect on what they have found (Petty et al. 2002). NFC relates to a deeper and broader information processing through greater investment of cognitive resources (Petty and Cacioppo 1986; von Stumm and Ackerman 2013), which has also been supported by neurophysiological studies (Mussel et al. 2016).

A recent meta‐analysis reported a small average correlation of *r* = 0.20 between NFC and academic achievement, which increased with grade level (Liu and Nesbit 2023). NFC shows incremental validity in explaining academic achievement over established predictors such as cognitive ability, openness, persistence, academic self‐concept, and intrinsic motivation (Lavrijsen et al. 2023; Strobel et al. 2024). Students with high NFC show more engagement when they expect a task to be challenging (Steinhart and Wyer 2009). In line with this finding, NFC predicts learning especially in more cognitively demanding learning environments (Colling et al. 2022). Individual differences in NFC are independent of students' gender, their socioeconomic status (SES), or cultural background (Bergold and Steinmayr 2023; Bertrams and Dickhäuser 2010; Colling et al. 2022; Preckel and Strobel 2017). NFC is usually assessed as a domain‐general construct. Keller et al. (2019) developed domain‐specific NFC scales and found that NFC showed strong domain‐generality and less domain‐specificity than academic self‐concept or academic interest.

Although NFC has been thoroughly studied in the context of academia, little is known about its development over time. Cacioppo et al. (1996) hypothesized that NFC develops through a recurring process of positive feedback and a sense of competence and satisfaction when successfully meeting cognitive challenges. In line with this assumption, Bergold and Steinmayr (2023) found that academic achievement positively predicted the development of NFC in 445 elementary‐school students after one year; their findings further indicated mean‐level stability and moderate retest stability (*r* = 0.56) of NFC over time. In a study with 5746 respondents who participated in a five‐wave online personality questionnaire, Bruinsma and Crutzen (2018) found that NFC increased between the ages of 16 and 24 years, remained fairly stable after the age of 25, and decreased in later years. A recent longitudinal study of 3409 adolescents assessed between Grades 7 and 12 found a latent rank‐order stability of NFC of *r* = 0.50 and individual differences in trajectories of NFC over time that could be partly explained by students' cognitive ability and home environment (i.e., cognitive stimulation and parental autonomy support; Lavrijsen et al. 2024).

### 1.2 Academic Interest

As a state, being interested in a certain situation means to experience joy and involvement, focused attention, and a sense of personal importance or value (situational interest; Hidi and Renninger 2006; Krapp 2002). As a trait, individual interest reflects an enduring disposition to engage with a specific content that can be stimulated in a certain situation (Ainley 2006). Academic interest refers to interest in content related to the academic field, for example, a specific school subject (e.g., mathematics), a specific content within a subject (e.g., geometry), or to a specific action (e.g., drawing geometric figures). It has a positive impact on student engagement in class (Ainley 2006) and has been described as a “positive energy” that facilitates learning (Harackiewicz and Knogler 2017, 334). Accordingly, academic interest is closely linked to an extensive (evolving) knowledge base in the topic of interest (Renninger 2000). Whether it is a state or a trait, academic interest is always linked to a specific object, action, or context because it is initially triggered and develops over time by individuals' interactions with their environment (“person‐object theory of interest”; Krapp 2002).

Academic interest and achievement show positive correlations of small to medium size (*r* = 0.16 to *r* = 0.32; Schiefele et al. 1992). Individuals are more interested in domains in which they are high achievers and have a high academic self‐concept (Denissen et al. 2007). The Programme of International Student Assessment (PISA) revealed that male students were more interested in mathematics (*d* = 0.22; OECD 2013) and female students were more interested in reading (*d* = −0.53; OECD 2003). Further, academic interests were found to be positively correlated with students' SES (mathematics: *d* = 0.15; OECD 2013; reading: *d* = 0.26; OECD 2003).

In their meta‐analysis on the development of motivational constructs, Scherrer and Preckel (2019) combined interest, intrinsic motivation, and flow under the term “intrinsic motivation” and found a small but steady mean‐level decline over the K‐12 school years (Glass's ∆ = −0.19, 95% CI [−0.25, −0.13] over an average of 1.66 years). Findings from Aschbacher et al. (2010) suggest that SES may influence the development of academic interest over time. Students from higher SES backgrounds were more likely to maintain a science interest in secondary school than students from lower SES backgrounds. Regarding gender, Renninger and Su (2019) conclude that there is stronger evidence for gender differences in the level of interest than in the trajectory of interest development over time.

## 2 Need for Cognition and Academic Interest

The interaction between the individual and the environment is an important distinguishing feature of academic interest in relation to NFC. While NFC is rather domain‐general and refers to the joy of “thinking per se” (Cacioppo and Petty 1982, 119), academic interest instead relates to specific content (Krapp 2002). One may have a somewhat general interest in learning or academics (Ainley et al. 2002), but past research has found that domain‐specific interests are only weakly related across domains (Nagy et al. 2006). Gogol et al. (2017) found considerably less domain‐general variability in academic interest for mathematics, French, and German as compared to domain‐specific variability (domain‐general: 21%–35%; domain‐specific: 65%–79%). Based on these findings, the authors suggest that domain‐general variability in interest should be interpreted as related to more general traits such as NFC rather than as general academic interest.

NFC and academic interests have similar nomological nets. Both are positively related to motivational variables in the learning context, with small to moderate relations to mastery goals (Day et al. 2007; Scherrer et al. 2020), cognitive effort (Keller et al. 2016; Trautwein et al. 2015), self‐regulated learning (Cazan and Indreica 2014; Soemer and Schiefele 2019), academic self‐concepts (Gogol et al. 2017; Keller et al. 2016), a deep approach to learning (Bråten et al. 2014; Cazan and Indreica 2014), and larger relations to curiosity (Bergold and Steinmayr 2023; Tang et al. 2022). For details, see the Table S1.

Murayama (2022) integrates both constructs in a reward‐learning framework of knowledge acquisition because NFC and academic interest support the process by which learning becomes rewarding in itself. In his Intellect framework, Mussel (2013) describes NFC and academic interest along the same motivational orientations of Seek and Conquer. Seek reflects processes that support approaching cognitively demanding situations, receptivity to these situations, and connection to positive affect. Conquer describes processes to engage, invest, and persist in the presence of challenges (Fleischhauer et al. 2010; Petty et al. 2002; Preckel and Strobel 2017). Despite these similarities in motivational orientations, NFC and interest can be distinguished in terms of preferences for cognitive activities. Whereas in the Intellect framework, NFC is characterized by a general preference for intensive thinking, problem solving, and analyzing complexity, interest is characterized by a preference for acquiring knowledge and skills, filling knowledge gaps, and learning to gain understanding and competencies related to the content of interest (Krapp 2002; Renninger et al. 2019; Tulis and Fulmer 2013). This knowledge and skill acquisition may be supported by the enjoyment of effortful thinking related to NFC.

Empirically, studies have revealed small to moderate correlations between NFC and academic interest (*r* = 0.27 to *r* = 0.62; Bråten et al. 2014; Feist 2012; Keller et al. 2019; Keller et al. 2016; Preckel 2014). Few studies have investigated their relations longitudinally. Bråten et al. (2014) found that earlier NFC predicted later situational interest in texts about science (β = 0.19). However, when individual interest was taken into account, NFC provided no incremental predictive value for situational interest beyond individual interest. Gottfried et al. (2017) found that academic interest in 9‐year olds predicted NFC later in life, but initial levels of NFC at age 9 were not assessed in this study. To the best of our knowledge, reciprocal relations between NFC and academic interest have not been investigated in previous studies.

### 2.1 Possible Links Between NFC and Academic Interest Over Time

The Four‐Phase Model of Interest Development by Hidi and Renninger (2006) describes the development from situational to individual interest within four phases: triggered situational interest, maintained situational interest, emerging individual interest, and well‐developed individual interest. (1) Triggered situational interest is characterized by selective attention to the content of interest and heightened affect. (2) Maintained situational interest is a phase in which the focus on the content of interest is sustained over an extended period, and individuals tend to exhibit positive feelings when engaging with the content, acquire knowledge about it, and assign value to the content. (3) Emerging individual interest represents the transition from the state of being interested to the trait individual interest. In this phase, the tendency to engage with the content of interest becomes increasingly autonomous, and individuals continue to acquire knowledge about it. (4) Finally, well‐developed individual interest describes the persistent motivation to revisit specific content of interest. Individuals repeatedly engage in tasks or challenges related to the topic of interest, are motivated to find creative solutions, and are persistent in doing so (Hidi and Renninger 2006; for a summary of empirical evidence, see Tang et al. 2022).

NFC and interest are possibly positively linked throughout these phases. Renninger et al. (2019) found that positive affect and autonomy moderated the triggering and maintaining of interest, and students with high NFC show higher levels of positive affect and autonomy in learning settings (von Stumm and Ackerman 2013). At the same time, experiencing particularly positive affect in challenging learning situations may benefit the development of NFC. Once interest is sustained over some time, learning about the content of interest becomes rewarding in itself, facilitating the search for information and the construction of a knowledge base (Murayama 2022; Renninger et al. 2019). This relation may be reinforced by an individual's motivation to invest cognitive resources (von Stumm and Ackerman 2013) and engage in learning opportunities (Cacioppo et al. 1996). Meanwhile, maintained situational interest may support the development of NFC by furthering engagement itself (Ainley 2006). More extensive knowledge may contribute to mastering cognitive challenges and a sense of competence (Murayama 2022), which in turn supports the development of interest (Renninger et al. 2019) and NFC (Cacioppo et al. 1996). Individuals with a well‐developed interest reengage with the content autonomously, which requires self‐regulation and perseverance (Renninger and Hidi 2016). Students with high NFC show better self‐regulation (Cazan and Indreica 2014) and more effort (Keller et al. 2016), which may assist them in maintaining focus on the content of interest. In line with this, NFC would support individuals to fully invest resources into acquiring knowledge about a content area of interest (von Stumm and Ackerman 2013). In conclusion, there are several plausible links between NFC and academic interests and their development. However, we lack empirical evidence for reciprocal effects in the development of NFC and academic interests.

## 3 The Present Study

The present study aims to gain insights into the joint development of NFC and academic interest by investigating reciprocal relations between the two constructs in secondary school students across three domains (i.e., mathematics, German, and English as a foreign language). Students either attended regular classes or special classes for the gifted at the same schools. Single studies have investigated the prediction of interest by NFC (Bråten et al. 2014) and vice versa (Gottfried et al. 2017). However, none have examined reciprocal relations over time. Based on the available cross‐sectional findings and our literature review of plausible relations in the development of academic interests and NFC reported above, we expected to find positive reciprocal relations between both constructs. Our hypotheses were as follows: Previous NFC positively predicts subsequent academic interest in mathematics, German, and English over time (Hypothesis 1). Previous academic interest in mathematics, German, and English positively predicts subsequent NFC over time (Hypothesis 2). We ran our analyses separately by academic achievement domain (i.e., mathematics, German, and English).

As NFC and academic interest are positively related to academic achievement (Liu and Nesbit 2023; Schiefele et al. 1992) and (are assumed to) develop in interaction with academic achievement (Cacioppo et al. 1996; Hidi and Renninger 2006), we ran our analyses with and without controlling for students' domain‐specific academic achievement.

Moreover, NFC and its development should generally be independent of students' gender and SES (Bergold and Steinmayr 2023; Bertrams and Dickhäuser 2010; Colling et al. 2022). However, there may be gender differences in academic interests in different domains (OECD 2003, 2013). In addition, students' SES may influence the development of their academic interest over time (Aschbacher et al. 2010). Students' SES is generally linked to their class type, especially because students from lower SES families are less likely to be identified for gifted programs (Hamilton et al. 2018). Finally, attending a gifted class might influence students' academic interest and NFC because these classes provide a more challenging academic curriculum and more options for independent and interest‐based studies (Schneider et al. 2014). Therefore, we additionally controlled our findings for a possible influence of gender, SES, and class type to explore alternative explanations for the cross‐lagged paths.

## 2 Method

### 2.1 Procedure

Our study presents a secondary data analysis with data stemming from the PULSS project (“Projekt für die Untersuchung des Lernens in der Sekundarstufe”), a longitudinal study of secondary school students in the German federal states of Bavaria and Baden‐Wuerttemberg. Data collection took place between 2008 and 2012. Students were assessed four times, at the beginning of Grade 5 (T1) and the end of Grade 5 (T2), the end of Grade 6 (T3), and in the middle of Grade 7 (T4). NFC was assessed on the first three measurement occasions. Academic interest and achievement in mathematics, German, and English were assessed at each of the four measurement occasions. The NFC and academic interest items were part of the same questionnaire, along with items assessing demographic information (i.e., age, gender, and first language). Parents reported their highest level of education in a separate parent questionnaire. Academic achievement was assessed with three separate standardized achievement tests for the different subjects. Trained research assistants conducted assessments of the students in their classrooms. All students participated voluntarily, and written informed consent was obtained from all parents. No experimental manipulations were performed, and all excluded data and analyzed variables are reported.

### 2.2 Participants

A total of 922 students were examined (41% female, age at T1 *M* = 10.63, SD = 0.55). All of them attended the highest track of the German secondary school system (academic track), also known as Gymnasium; 639 students attended 26 regular classes, and 283 students attended 14 gifted classes, which were offered as full‐time ability grouping classes within the highest track. Tracking began in Grade 5 with the start of secondary school. In the German school system, the highest track (academic track) prepares students for university enrollment with a high school diploma (“Abitur”), compared to the non‐academic tracks: the middle track (secondary school level I certificate) and the lowest track (certificate of secondary education), which prepare students for vocational qualification. Most students had a higher SES, with 63% of parents having a university degree, 16% the Abitur (high school diploma), 19% a secondary school level I certificate, and 2% a certificate of secondary education. Most of the students' first language was German (90%), followed by Russian, English, Turkish, or other. Students with a first language other than German reported speaking German for at least one year (up to twelve years; *n* = 117; *M* = 8.31, SD = 2.12). Students' first foreign language in school was mostly English (76%) or Latin (24%). As the present study represents a secondary data analysis, we were constrained in terms of the demographic characteristics of the sample.

### 2.3 Measures

#### 2.3.1 Demographic Variables

Students reported their age, gender, and first language. Gender was coded dichotomously (i.e., 0 = male, 1 = female). The students' parents reported their highest level of education. We used the highest level of education attained by either parent as an indicator of student SES (1 = no school degree, 2 = certificate of secondary education, 3 = secondary school Level I certificate, 4 = high school diploma, 5 = university degree, 6 = doctorate degree).

#### 2.3.2 Need for Cognition

NFC was assessed using the NFC‐Teens scale (Preckel 2014, 2016), which was adapted from Cacioppo and Petty's (1982) adult NFC scale for adolescents and the German language. A sample item is “I like to do tasks that require me to think a lot.” (translation by the authors). Answers were given on a five‐point Likert scale (1 = *strongly disagree* to 5 = *strongly agree*). Previous studies have provided support for the construct validity of the NFC‐Teens scale, including finding a latent correlation of *r* = 0.99 between the general factors of the NFC‐Teens scale and another NFC scale within a sample of Grade 9 students in Luxembourg (*N* = 443; Keller et al. 2019). In addition, there is evidence to confirm the factorial structure of the NFC‐Teens scale, as well as its external validity with respect to academic achievement, academic interest, and goal orientation (Preckel 2016). Preckel (2014) found effects of positive and negative item wording (e.g., correlations with academic achievement and cognitive ability). To avoid these confounding factors, we used only the 11 positively worded items (out of 19). The correlation of the shortened scale with the total scale was *r* = 0.91. Its internal consistency at the four measurement points ranged from McDonald's ω = 0.89 to ω = 0.92.

#### 2.3.3 Academic Interest

Academic interest in mathematics, German, and English was measured using a three‐item scale for each domain. Items stem from the Self‐Regulation Questionnaire (Ryan and Connell 1989). The wording was identical across domains, differing only in the specific domain to which the item referred: “I do my mathematics/German/English homework because I enjoy this subject”, “I participate in mathematics/German/English class because I have a strong interest in this subject”, and “I try hard in mathematics/German/English class because this subject interests me.” (translation by the authors). Answers were given on a five‐point Likert scale. This assessment represents individual academic interest in the respective domains, although it is possible that students rated a situational component of their interest as well. However, Renninger and Su (2019) highlight that students may encounter difficulty in identifying a triggered interest (an early phase, situational interest) through self‐reports. The internal consistency in mathematics, German, and English at the four measurement points ranged from McDonald's ω = 0.68 to ω = 0.90, ω = 0.88 to ω = 0.89, and ω = 0.89 to ω = 0.91, respectively.

#### 2.3.4 Achievement

Standardized achievement tests with four progressively difficult test versions were administered to account for the increasing knowledge and achievement over time.

##### 2.3.4.1 Mathematics

Mathematics achievement was assessed with the German Mathematics Test for Grade 5/6 (“Deutscher Mathematiktest für fünfte/sechste Klassen” [DEMAT 5+/6+]; Götz et al. 2013a, 2013b). The tests are aligned with the school curricula and cover arithmetic, algebra, geometry, and word problems. There were no indications for ceiling effects (skewness values between −0.08 and 0.25). The reliabilities of the person parameters were acceptable to good (weighted likelihood estimate reliability [WLE‐Rel] between 0.77 and 0.88; see Table S2).

##### 2.3.4.2 German

German language achievement was first assessed with the predominantly speed‐based Reading Speed and Comprehension Test for Grades 5 through 12+ (“Lesegeschwindigkeits‐ und Verständnistest für die Klassen 5–12” [LGVT 5–12+]; Schneider et al. 2017). The students were given 4 min to read a text and their word count was measured. To encourage conscientious reading, students were asked to select the word that best fit the context from a set of options at several points in time. This reading speed task was applied at T1, T2, and T4. Furthermore, two reading comprehension tests were used. For T1 and T2, the Frankfurter Reading Comprehension Test for Grades 5 through 6 was used (“Frankfurter Leseverständnistest für 5. und 6. Klassen” [FLVT 5–6]; Souvignier et al. 2008). Students were given 15 min to complete the test. As several students had completed the test at the end of Grade 6, a second test, the Reading Test Battery for Grades 8 through 9 (“Lesetestbatterie für die Klassenstufen 8‐9” [LESEN 8–9]; Bäuerlein et al. 2012), was used for the remaining students for T3 and T4. Both tests required students to answer multiple‐choice questions after reading a fictional and a nonfiction text. There were no indications for ceiling effects (skewness values between −0.21 and 0.22). The reliabilities of the person parameters were good (WLE‐Rel between 0.82 and 0.87; see Table S2).

##### 2.3.4.3 English

English language achievement was assessed by four versions of the English Test for Grades 5 to 7 (Harder and Ziegler 2009). The content was based on the English curricula and included vocabulary, comprehension, and grammar tasks. There were no indications for ceiling effects (skewness values between 0.19 and 0.58). The reliabilities of the person parameters were good (WLE‐Rel between 0.85 and 0.89; see Table S2).

### 2.4 Data Analysis

#### 2.4.1 Achievement Test Score Equation

To scale achievement across the four test versions, we applied a chain‐linking procedure using a unidimensional Rasch model within each domain. Item and person parameters were estimated (for details, see Achievement Test Equating in the online supplemental material).

#### 2.4.2 Model Estimation

Data analyses were conducted with Mplus 8.7 (Muthén and Muthén 1998–2021). We used maximum likelihood estimation with robust standard errors. The full information maximum likelihood approach implemented in Mplus was used to handle missing data. Type is complex was specified to account for the nested data structure. The data yielded 40 clusters (school classes). Effect coding was applied by constraining the average factor loadings to 1 and the average item intercepts to 0. Thus, the latent factor means represent the average mean of the indicators weighted by their factor loadings (Little et al. 2007). Model fit was examined using the chi‐squared statistic, the comparative fit index (CFI), the root mean square error of approximation (RMSEA), and the standardized root mean square residual (SRMR; Hu and Bentler 1999). To ensure that variables were measured comparably over time, we tested for measurement invariance of the NFC and academic interest measures over time. Based on Chen's (2007) recommendation, we considered a restricted model as having an acceptable deterioration in model fit compared to the unrestricted model when the difference in CFI did not exceed 0.01 (ΔCFI ≤ 0.01).

#### 2.4.3 Hypothesis Testing

**FIGURE 1:**
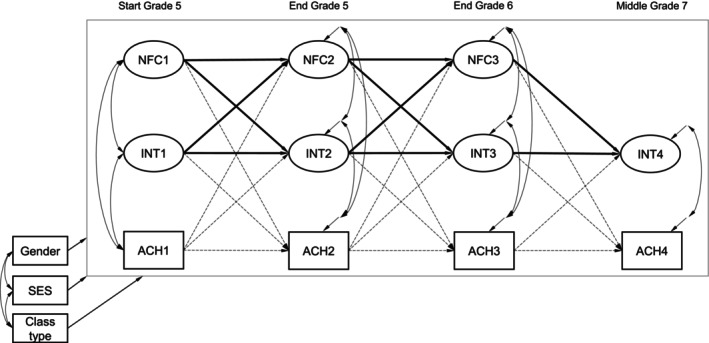
Schematic autoregressive cross‐lagged panel models with control variables.

The analyses are based on a confirmatory approach. Autoregressive cross‐lagged panel models (CLPM) were conducted over the four measurement occasions in a two‐step approach. In the first step, in Model 1, the cross‐lagged paths between NFC and academic interest were investigated (see Figure 1). For Hypothesis 1, we examined the regression weights of NFC at TX‐1 predicting academic interest at TX. For Hypothesis 2, we examined the regression weights of academic interest at TX‐1 predicting NFC at TX. To control for academic achievement, additional cross‐lagged paths to domain‐specific academic achievement per measurement occasion were added in Model 2 (see dotted arrows in Figure 1). In a second step, we replicated Model 1 and Model 2 controlling for students' gender, SES, and class type to test the robustness of our findings (i.e., the regression of all variables at each measurement point on the control variables).

## 3 Results

Descriptive statistics for the variables investigated are presented in Table 1. The intraclass correlation coefficients (ICCs) were higher for achievement than for academic interest or NFC. This indicates that there was greater variability between classes in achievement than in academic interest or NFC. Latent correlations between NFC and academic interest ranged from 0.08 to 0.59 (for all latent bivariate correlations, see Table S3). We observed decreases in the mean levels of students' NFC and academic interest over time. Between T3 and T4, reflecting a half‐year rather than a full‐year interval, academic interest remained fairly stable in all domains (model estimated latent means, see Table 1). Achievement test scores increased over time.

**TABLE 1:** Descriptive statistics for need for cognition, academic interest, and achievement by domain.

Measure	Start of Grade 5 (T1)				End of Grade 5 (T2)				End of Grade 6 (T3)				Middle of Grade 7 (T4)			
	M (SE)	Skewness (SE)	Kurtosis (SE)	ICC	M (SE)	Skewness (SE)	Kurtosis (SE)	ICC	M (SE)	Skewness (SE)	Kurtosis (SE)	ICC	M (SE)	Skewness (SE)	Kurtosis (SE)	ICC
NFC	3.56 (0.04)	−0.21 (0.08)	−0.29 (0.17)	0.04	3.42 (0.05)	0.00 (0.08)	−0.37 (0.17)	0.10	3.31 (0.05)	0.00 (0.09)	−0.05 (0.18)	0.13				
INT_M	4.12 (0.04)	−1.05 (0.08)	0.43 (0.17)	0.04	3.73 (0.07)	−0.57 (0.09)	−0.47 (0.17)	0.10	3.46 (0.07)	−0.36 (0.09)	−0.67 (0.18)	0.13	3.50 (0.06)	3.67 (0.09)	53.55 (0.19)	0.07
INT_G	3.71 (0.05)	−0.06 (0.08)	−0.17 (0.17)	0.05	3.34 (0.07)	−0.27 (0.09)	−0.69 (0.17)	0.11	3.11 (0.08)	−0.18 (0.09)	−0.61 (0.18)	0.17	3.09 (0.07)	−0.12 (0.09)	−0.59 (0.19)	0.12
INT_E	4.23 (0.04)	−1.31 (0.08)	1.40 (0.17)	0.04	3.92 (0.05)	−0.79 (0.09)	−0.06 (0.17)	0.04	3.68 (0.06)	−0.45 (0.09)	−0.35 (0.18)	0.09	3.52 (0.06)	−0.46 (0.09)	−0.29 (0.19)	0.10
ACH_M	−0.58 (0.09)	0.14 (0.08)	0.19 (0.17)	0.28	−0.22 (0.11)	0.19 (0.08)	0.37 (0.17)	0.38	0.11 (0.12)	−0.08 (0.09)	0.56 (0.18)	0.31	0.38 (0.10)	0.25 (0.09)	0.41 (0.19)	0.32
ACH_G	−0.01 (0.07)	−0.21 (0.08)	0.43 (0.17)	0.20	0.56 (0.07)	0.22 (0.08)	1.82 (0.17)	0.23	1.25 (0.09)	−0.17 (0.09)	0.52 (0.18)	0.29	1.79 (0.08)	−0.17 (0.09)	2.88 (0.19)	0.24
ACH_E	0.11 (0.12)	0.19 (0.10)	2.28 (0.19)	0.27	1.82 (0.12)	0.24 (0.10)	0.57 (0.19)	0.35	2.49 (0.12)	0.58 (0.10)	0.92 (0.21)	0.31	3.29 (0.12)	0.41 (0.11)	0.47 (0.22)	0.29

*Note:* Latent means and standard errors are model estimated.

Abbreviations: ACH, academic achievement; E, English; G, German; ICC, intraclass correlation coefficient; INT, academic interest; M, mathematics; NFC, need for cognition.

### 3.1 Measurement Invariance Testing

Results of measurement invariance tests for the NFC and academic interest measures are presented in Table S4. Scalar measurement invariance held for all academic interest measures (∆CFI = 0.001 to ∆CFI = 0.003 compared to the metric models). For NFC, we set free the intercepts for Items 1, 3, and 9 to achieve an adequate model fit for (partial) scalar measurement invariance (∆CFI = 0.007 compared to the metric model).

### 3.2 Autoregressive Cross‐Lagged Panel Modeling

**FIGURE 2:**
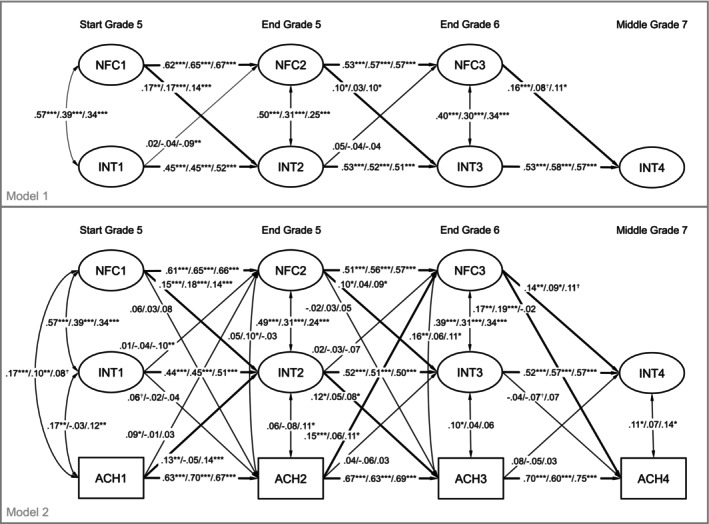
Results of the autoregressive cross‐lagged panel models 1 and 2 without controlling for gender, SES, and class type.

The CLPMs yielded adequate model fit indices (CFI > 0.94, RMSEA < 0.06, SRMR < 0.06; see Table 2). Figure 2 presents the standardized coefficients for Model 1 and Model 2 without gender, SES, and class type as control variables. Stabilities (autoregressive effects) were somewhat higher for NFC than for academic interest between T1 and T2 but comparable afterwards and of medium size. For Model 1, in mathematics and English, previous NFC was positively related to subsequent academic interest over all measurement points. In German, previous NFC was only positively related to subsequent academic interest at T2. Academic interest was not significantly related to subsequent NFC (with one exception: previous interest in English was negatively related to NFC at T2). Findings did not change substantially when controlling for academic achievement in Model 2 (e.g., previous NFC was positively related to subsequent academic interest but not vice versa with the exception of a negative prediction of NFC at T2 by previous interest in English). Compared to Model 1 without academic achievement included, two regression paths between NFC at T3 and German interest at T4 and English interest at T4 changed in significance (German: *p* = 0.072 to *p* = 0.040; English: *p* = 0.045 to *p* = 0.059). We expect that the reported significant results are not due to sampling error, because we examined a sufficiently large dataset for structural equation modeling and model fits indicate an adequate fit to the data.

**TABLE 2:** Model fit for model 1 and model 2 (achievement added) by domain.

Model	*χ* ^2^	df	*p*	SCF	CFI	RMSEA [90% CI]	SRMR
Model 1							
Mathematics	1765.473	927	< 0.001	1.113	0.950	0.031 [0.029, 0.034]	0.046
German	1759.579	927	< 0.001	1.141	0.953	0.031 [0.029, 0.033]	0.054
English	1762.231	927	< 0.001	1.153	0.952	0.031 [0.029, 0.034]	0.050
Model 2							
Mathematics	2218.711	1092	< 0.001	1.095	0.941	0.034 [0.032, 0.036]	0.048
German	2206.957	1092	< 0.001	1.122	0.942	0.033 [0.031, 0.035]	0.054
English	2086.695	1092	< 0.001	1.125	0.948	0.031 [0.029, 0.034]	0.049

Abbreviations: SCF, scaling correction factor; CFI, comparative fit index; RMSEA, root mean square error of approximation; SRMR, standardized root mean square residual.

When including gender, SES, and class type as control variables in Models 1 and 2, findings did not change substantially (see Figure S1, Tables S6 and S7). The total number of significant paths at *p* < 0.05 between NFC and academic interest changed from 8 to 7 after including the control variables; at the *p* < 0.10 level, they remained unchanged (at 9). Higher levels of NFC were related to a more positive development of academic interest in all three domains. Academic interest, on the other hand, was not significantly related to subsequent NFC with one exception: a higher interest in English at T1 was related to lower levels of NFC over time. Students' gender predicted NFC (T1; higher for males), mathematics interest (T1 and T3; higher for males) and mathematics achievement (T1; higher for males) as well as German interest (T1; higher for females), German achievement (T4; higher for females), and English achievement (T1 and T2; higher for females). SES negatively predicted English interest at T1 (higher for lower SES). Class type positively predicted NFC (higher for gifted classes), interest in German (higher for regular classes), and academic achievement (higher for gifted classes), especially at T1 but also throughout.

## 4 Discussion

We investigated reciprocal relations between NFC and academic interest over two and a half years of secondary school education across three domains: mathematics, German, and English. We found that higher NFC was related to a more positive development of academic interest, supporting Hypothesis 1. However, and not in support of Hypothesis 2, previous academic interest was largely unrelated to the development of NFC. Notably, these results were rather consistent across the different academic domains and remained stable when controlling for students' academic achievement, gender, SES, and class type.

### 4.1 Need for Cognition and Academic Interest Development

According to Hidi and Renninger (2006), “The potential for interest is in the person but the content and the environment define the direction of interest and contribute to its development” (p. 112). In addition, according to Renninger et al. (2019), “relatively little is understood about the process that results in triggering and then maintaining the development of interest” (p. 1). Our findings characterize NFC as part of a person's potential for academic interest that may help trigger and then maintain the development of interest. NFC seems to contribute to a learning context that is particularly beneficial for the development of academic interest possibly by interacting with the content of interest via positive affect, information search, knowledge acquisition, effort, and persistence. There are several starting points where NFC may play a role in the development of academic interest, including becoming interested in the first place (i.e., triggered situational interest). Expanding the findings from Bråten et al. (2014), we found that NFC also predicted a more stable academic interest and not exclusively a situational academic interest. Students with higher NFC benefit in school in terms of their academic interest development, possibly because complex tasks in students' content areas of interest are more likely to evoke high engagement and resource investment (Fulmer et al. 2015). Renninger and Hidi (2016) propose that interest development is indicated particularly by frequent, autonomous, and intensive engagement, as well as knowledge acquisition, and NFC is positively related to all of these processes. The Intelligence‐as‐Process, Personality, Interest, and Intelligence‐as‐Knowledge (PPIK) theory of intellectual development (Ackerman 1996) similarly argues that NFC enhances cognitive engagement and persistence, thereby supporting the development of academic interest. Notably, our data do not allow us to make any statements about the individual phases of academic interest development or the specific functional relations between NFC and academic interest over time. There is a need for more research in this area, including experiments and finer‐grained data collection.

Regarding the influence of academic interest in the development of NFC, we assumed that academic interest would contribute to a learning context in which individuals are encouraged to think intensively and solve problems by thinking, thereby fostering their NFC development. However, against our expectations, interest was unrelated to the development of NFC in this study with one exception, where interest in English at the first measurement point was negatively related to later NFC. At the later measurement points, which only reflect the change in constructs, we did not find this association. Therefore, this finding should not be over‐interpreted. It may be explained by third variables not assessed and controlled for that only affected the first measurement point (e.g., experience with English as a foreign language in elementary school).

NFC represents a general motivation for information search and reflection (Petty et al. 2002). Being interested in a particular school subject helps to explore, learn, and understand a specific topic (Renninger and Hidi 2016), but may not provide enough opportunities to influence the development of this personality trait. According to the reward‐learning framework of knowledge acquisition (Murayama 2022), learning about a specific content becomes rewarding so that acquired knowledge motivates further learning. The authors suggest that this “self‐boosting property” (p. 182) may explain the relation between learning and the development of interest and personality traits such as curiosity. However, we only found a positive relation of NFC for the development of academic interest but no common development or reciprocal relations between NFC and academic interest. If we assume that NFC partly develops from mastering cognitive challenges as proposed by Cacioppo et al. (1996), it may be possible that the classes in Grade 5 to Grade 7 were not challenging enough so that students with a well‐developed academic interest could not create enough opportunities for deep thinking and mastering challenges to support their NFC development. However, our sample included students from gifted classes that provided a more challenging academic curriculum and more options for independent and interest‐based studies (Schneider et al. 2014). Therefore, this explanation seems less plausible. More research is needed that takes into account theoretical considerations and empirical evidence on the development of NFC (Aerts et al. 2024).

### 4.2 Limitations and Implications for Future Research

Some limitations need to be considered in the interpretation of our findings. First, although we analyzed a large sample of students, the present study is a secondary analysis of previously collected data. Therefore, we were limited in the time frame of two and a half years of students' educational careers, the demographic characteristics of our sample, the assessment schedule, and the study variables. Academic interests may be more developed and provide higher predictive power in students of higher grades than in this analysis, where students had just transitioned to secondary school at the first measurement point. We have seen a decline in academic interests during this period. However, this does not necessarily mean that this trend will continue. The decline may have been influenced by experiences specific to the students' classes at the time. For example, changes in curricula or teachers could lead to different trends. Furthermore, the observed decline could also be explained by a general decline in interest over the course of the school year (Scherrer and Preckel 2019). Taken together, the observed decline does not necessarily provide insight into students' future academic aspirations, such as their intention to pursue further and higher education in the three domains under study.

In addition, the schools were exclusively German high‐track schools that prepare students for academic higher education, which means that our findings cannot be generalized to lower‐track schools or other countries without further investigation. Colling et al. (2022) found a stronger relation between NFC and learning outcomes in more cognitively demanding learning environments, which might also apply to academic interest. That is, it cannot be excluded that in less cognitively demanding learning environments that offer less opportunities to experience and enjoy cognitive challenge there is a weaker relation between NFC and academic interest. On the other hand, our sample was more homogeneous than a student sample that is composed of students from more diverse school types. For example, most of the students in our sample were from higher SES backgrounds, which is positively related to students' academic interest and its development (Aschbacher et al. 2010). This homogeneity may have resulted in a limited variance in our data, which could lead to an underestimation of the strength of the relations. Therefore, our analyses need to be replicated with more diverse samples of students.

Conceptually, our focus has been on the relations between NFC and academic interest over time on a broader scale, considering multiple subject domains. Given our analyses, we cannot make any statements about academic interest development at the intraindividual level. Additionally, our data do not allow us to separate situational from individual academic interest. Future studies should examine whether NFC predominantly relates to a more individual academic interest or whether there is also a relation to short‐term situational academic interest in certain learning contexts. To further clarify the processes of triggering and maintaining academic interest, it would also be relevant to examine the specific phase(s) in which NFC becomes a predictor of academic interest. A more process‐oriented study of the development with additional and more frequent measurement points would be needed to assess situational academic interest and identify corresponding developmental phases. There are indications that a connecting variable between NFC and academic interest could be curiosity. Curiosity describes the need or motivation to learn new information in the face of a knowledge deficit (Loewenstein 1994). Curiosity is strongly related to both NFC and interest (see Table S1). However, previous studies have mainly focused on the relation between curiosity and either NFC or academic interest. Aerts et al. (2024) describe similarities between curiosity and NFC as investment traits and differences in NFC being focused on cognitive activity and curiosity revolving around a gap in the knowledge. Tang et al. (2022) describe similarities in the conceptualization of curiosity and interest as motivational variables and differences in, for example, their neurological pathways. What is missing is an integration between these research fields. It is conceivable that there is a developmental path between the cognitive activity‐oriented NFC, the curiosity to discover and fill knowledge gaps, and a developing interest in the subject being studied. Therefore, future studies should aim to integrate findings on the relations between NFC, curiosity, and academic interest in order to contribute to a comprehensive understanding of the role of NFC in the development of academic interest.

Finally, we cannot rule out the influence of other third variables, such as academic self‐concepts that are positively related to both, NFC and academic interests. However, we controlled for academic achievement, which shares considerable variability with academic competence perceptions such as academic self‐concept. In addition, a common influence of a third variable on interest and NFC would have affected both variables, but we quite consistently found a positive relation of NFC and subsequent academic interest and no positive relation of academic interest and subsequent NFC.

### 4.3 Practical Implications: Promoting Academic Interest and NFC Development

In line with meta‐analytic findings on intrinsic motivation (Scherrer and Preckel 2019) and a recent study examining changes in secondary school students' NFC (Lavrijsen et al. 2024), we found that academic interest and NFC decreased over time. Since both constructs are beneficial to students' academic development, our findings suggest that attention should be paid to students' academic interest and NFC development and variables that influence their trajectories over time. Overall, our findings cannot be the basis for causal interpretations. However, we found a consistent relation between NFC and the development of academic interest. This leads us to consider a few tentative implications for practice.

Harackiewicz and Knogler (2017) propose that the development of academic interest can be supported by appreciating individual learner characteristics, which may include an already developed interest. Building on the results of this study, we would argue that this can also apply to NFC. Our findings suggest that it may be useful to measure students' NFC to find out which students with lower NFC may need more support in developing academic interests. To implement this, self‐report measures for NFC are available for students from elementary to secondary school (e.g., Preckel 2014; Preckel and Strobel 2017). Meta‐analytic evidence supports the notion that school‐based interventions can be effective in promoting students' motivation, including academic interest (*d* = 0.49, 95% CI [0.43, 0.56]; Lazowski and Hulleman 2016). For example, providing students with hands‐on activities related to reading, that allow them to work with an object, conduct experiments, or undertake projects, can promote the development of their individual interest in reading (Guthrie et al. 2006). In addition, Hulleman and Harackiewicz (2009) showed in a randomized control trial that students' interest in science increased when the students themselves identified the personal relevance of the topic being discussed in science class, especially for students who did not think success was likely.

Further, interventions could focus on promoting NFC development, which in turn may promote the development of academic interest. Students benefit from shaping the learning context in a way that adequately challenges them. As demonstrated by Lavrijsen et al. (2021), especially for students with high NFC, engagement and interest in class depend on being adequately challenged. Adding to that, Aerts et al. (2024) outline specific approaches in promoting NFC development, one of which is optimal challenge. When students are optimally challenged, they are more likely to succeed. In addition, compared to simple tasks, they must work hard and invest cognitive resources, which stimulates enjoyment and provides more opportunities to learn and believe in their abilities. Aerts et al. (2024) also note that providing optimal challenge in the classroom, as a characteristic of the learning environment may not be sufficient in promoting NFC development. Instead, the extent to which students view cognitive challenges as worthwhile, difficult to achieve, rewarding, and enjoyable may shape how they approach and enjoy challenging situations. These appraisals can be supported by a teachers' enthusiastic manner as well as recognizing and providing feedback on the task value and students' efforts. In addition, teachers can specifically support their students in mastering cognitive challenges by structuring tasks, providing individualized instruction tailored to students' needs, and by providing feedback on students' solutions in the learning process (for details and additional approaches, see Aerts et al. 2024).

Based on the present findings, interventions that promote academic interest may not be effective in promoting the development of NFC, but fostering NFC may be effective in promoting the development of interest—and therefore engagement—in school. However, to shed light on this issue, we need stronger evidence from multivariate studies with multiple measurement points of high frequency that also include starting points for the promotion of NFC and academic interest. For example, autonomy support from parents and teachers has been examined as a predictor of students' academic interests or intrinsic motivation and NFC. Engler and Westphal (2024) found that teacher autonomy support was associated with a more positive trajectory of academic interests, and a meta‐analysis by Bureau et al. (2022) found that teacher autonomy support predicted student intrinsic motivation, whereas parental autonomy support showed only weak associations with student motivation. However, Lavrijsen et al. (2024) found that parental autonomy support was associated with a higher NFC trajectory in students (i.e., high and stable NFC levels over time). Therefore, parental autonomy support may be indirectly related to student motivation through student NFC. These assumptions are rather speculative at this point, but they open up new avenues for research and practice to support students' motivational development.

## 5 Conclusion

Our study provides insights into the relation between NFC and academic interest over a period of two and a half years of secondary school education. Higher levels of NFC were related to a more positive development of academic interest, while previous academic interest mostly did not relate to the development of NFC. For the most part, the same relations were also observed after controlling for students' achievement, class type, and demographic characteristics. The results suggest that promoting students' NFC may be beneficial to the development of their academic interest. However, there is a lack of knowledge about NFC's development and ways to promote it (but see Aerts et al. 2024). Positive experiences in highly cognitively demanding situations may work towards the basis for developing motivation (Bergold and Steinmayr 2023; Lavrijsen et al. 2023). Promoting interest development in the academic context is crucial, especially considering the general decline in interest over time (Scherrer and Preckel 2019). Our study emphasizes the importance of appreciating individual learner characteristics in school. Students with low NFC may face greater challenges in developing academic interests. For these students, individualized attention may be instrumental in triggering and maintaining academic interest in school. The findings warrant further exploration of the role of NFC in academic interest development and in educational contexts in general.

## Author Contributions

**Julia Matthes:** conceptualization, methodology, formal analysis, writing – original draft, writing – review and editing, visualization. **Vsevolod Scherrer:** methodology, formal analysis, writing – review and editing. **Franzis Preckel:** conceptualization, methodology, writing – original draft, writing – review and editing.

## Supporting information


Data S1.

## Data Availability

The data necessary to reproduce the analyses presented here are not publicly accessible. The analytic code necessary to reproduce the analyses presented in this paper is publicly accessible. Code is available in the online supplemental material. The materials necessary to attempt to replicate the findings presented here are available on request from the last author (preckel@uni-trier.de). The analyses presented here were not preregistered.
