# Supplementary material for: Need for Cognition Predicts Academic Interest Development but Not the Other Way Around: A Longitudinal Study of Secondary School Students
Source: Child Dev. 2025 Jun 4;96(5):1675–87. doi: 10.1111/cdev.14262 (PMC12379847; doi:10.1111/cdev.14262)
Supplement: Supplementary file 1 — Data S1. [file CDEV-96-1675-s001.docx]

Online Supplemental Material

Conceptual Overlap Between Need for Cognition and Academic Interest

**Table S1**

*Correlations Between Need for Cognition and Academic Interest with Motivational and Cognitive Variables in the Learning Context*

| Variables | Correlation with NFC | Correlation with academic interest |
| --- | --- | --- |
| Mastery goals | *r* = .21 to *r* = .31 (German)^a^  *r* = .23 to *r* = .34 (mathematics)^a^  *r* = .25^b^ | *r* = .44^c^ |
| Cognitive effort | *r* = .13^d^  *r* = .42/*r* = .45^e^ | *r* = .26^f^ |
| Academic self-concepts | *r* = .40/*r* = .42/*r* = .49 (different self-concept measures)^e^  *r* = .41^g^ *r* = .45^h^ | *r* = .60/*r* = .67/*r* = .39/*r* = .54 (different samples)^i^ |
| Self-regulated learning | *r* = .33^j^ | *r* = -.36 (with voluntary mind wandering)^k^ |
| Deep approach to learning | *r* = .35^l^  *r* = .53^j^  *r* = .78^m^ | *r* = .27 (individual academic interest)^l^  *r* = .44 (situational academic interest)^l^  *r* = .58^n^ |
| Curiosity | *r* = .51/*r* = .70 (interest-type curiosity T1/T2 one year later)^o^ *r* = .58/*r* = .59 (deficit-type curiosity T1/T2 one year later)^o^  *r* = .57^p^ | *r* = .53^q^ |

Note. ^a^ Preckel (2014), ^b^ Day et al. (2007), ^c^ Scherrer et al. (2020), ^d^ Kramer et al. (2021), ^e^ Keller et al. (2016),
^f^ Trautwein et al. (2015), ^g^ Luong et al. (2017), ^h^ Dickhäuser & Reinhard (2010), ^i^ Gogol et al. (2017), ^j^ Cazan & Indreica (2014), ^k^ Soemer & Schiefele (2019), ^l^ Bråten et al. (2014), ^m^ Evans et al. (2003), ^n^ Bächtold et al. (2023), ^o^ Bergold & Steinmayr (2023), ^p^ Olson et al. (1984), ^q^ Tang et al. (2022).

Achievement Test Equating

Mathematics. In order to establish a common metric across the measurement points, a chain-linking procedure was employed under the common item equivalent groups design (mean-mean equating; Kolen & Brennan, 2014) on the basis of a one-dimensional Rasch model (Embretson & Reise, 2000). Common items were considered equally. We estimated item and person parameters with ConQuest 2.0 (Wu et al., 2007) and weighted maximum likelihood estimation (WLE; Warm, 1989, see Table S2). The values obtained served as fixed values for common items between T1 and T2. The remaining parameters for T2 were estimated freely to include all available items in the estimation of the person parameters for T2. The same procedure was applied for T3 and T4, utilizing the item parameters of the previous measurement occasion as a reference.

German and English. In order to establish a common metric across the measurement points, a chain-linking procedure was employed under the common item equivalent groups design (mean-mean equating; Kolen & Brennan, 2014). The basis was a one-dimensional Rasch model (R package TAM [Test analysis modules]; Robitzsch et al., 2019) estimating item and person parameters (WLE; Warm, 1989; see Table S2). Regarding German achievement, the values obtained were employed as fixed values for common items between T1 and T2. The remaining parameters for T2 were estimated freely. Consequently, all available items were included in the estimation of person parameters for T2. The tests used at the second and third measurement occasions did not share any common items. Therefore, the items were linked as follows: T1 to T2, T2 to T4, and T4 to T3. For English achievement, the values obtained served as fixed values for common items between T1 and T2. The remaining parameters for T2 were estimated freely to include all available items in the estimation of the person parameters for T2. The same procedure was applied for T3 and T4, utilizing the item parameters of the previous measurement occasion as a reference.

**Table S2**

*Common-Item Design for Equating, Item Parameters, and Reliability of Person Parameters of the Linked Achievement Test Versions (Tests 1 to 4)*

| Test Version | Test 1 | Test 2 | Test 3 | Test 4 | *N* | IP min | IP max | WLE-Rel |
| --- | --- | --- | --- | --- | --- | --- | --- | --- |
| Mathematics |  |  |  |  |  |  |  |  |
| Test 1 | 26 |  |  |  | 857 | -2.34 | 1.22 | .77 |
| Test 2 | 17 | 31 |  |  | 997 | -2.19 | 1.66 | .84 |
| Test 3 | 9 | 11 | 37 |  | 899 | -1.56 | 3.74 | .88 |
| Test 4 | 6 | 10 | 17 | 39 | 804 | -1.51 | 2.66 | .86 |
| German |  |  |  |  |  |  |  |  |
| Test 1 | 52 |  |  |  | 877 | -3.14 | 6.40 | .84 |
| Test 2 | 40 | 54 |  |  | 992 | -3.14 | 5.02 | .82 |
| Test 3 |  |  | 38 |  | 761 | -1.18 | 4.55 | .87 |
| Test 4 |  | 6 | 34 | 58 | 817 | -0.92 | 3.02 | .82 |
| English |  |  |  |  |  |  |  |  |
| Test 1 | 40 |  |  |  | 653 | -2.92 | 3.05 | .89 |
| Test 2 | 4 | 36 |  |  | 761 | -0.94 | 3.61 | .87 |
| Test 3 |  | 7 | 33 |  | 680 | -0.08 | 5.20 | .85 |
| Test 4 |  |  | 9 | 36 | 602 | -0.08 | 5.01 | .87 |

*Note.* Sum of items per test version in the diagonal. *N* = number of participants for parameter estimation; IP min = minimum value of item parameters; IP max = maximum value of item parameters; WLE-Rel = reliability of weighted likelihood estimate (WLE) person parameters. The tests were linked including an additional set of data, which we did not include in our analyses for the following reason: At this school, students did not participate in the first measurement occasion.

Intercorrelation of Measures

Table S3

Bivariate Latent Correlations of the Investigated Variables

| Variable | NFC1 | NFC2 | NFC3 | INT_M1 | INT_M2 | INT_M3 | INT_M4 | INT_G1 | INT_G2 | INT_G3 | INT_G4 | INT_E1 | INT_E2 | INT_E3 | INT_E4 | ACH_M1 | ACH_M2 | ACH_M3 | ACH_M4 | ACH_G1 | ACH_G2 | ACH_G3 | ACH_G4 | ACH_E1 | ACH_E2 | ACH_E3 | ACH_E4 | Gender | SES | Class type |
| --- | --- | --- | --- | --- | --- | --- | --- | --- | --- | --- | --- | --- | --- | --- | --- | --- | --- | --- | --- | --- | --- | --- | --- | --- | --- | --- | --- | --- | --- | --- |
| NFC1 | 1 |  |  |  |  |  |  |  |  |  |  |  |  |  |  |  |  |  |  |  |  |  |  |  |  |  |  |  |  |  |
| NFC2 | .63^***^ | 1 |  |  |  |  |  |  |  |  |  |  |  |  |  |  |  |  |  |  |  |  |  |  |  |  |  |  |  |  |
| NFC3 | .41^***^ | .55^***^ | 1 |  |  |  |  |  |  |  |  |  |  |  |  |  |  |  |  |  |  |  |  |  |  |  |  |  |  |  |
| INT_M1 | **.57^***^** | .37^***^ | .28^***^ | 1 |  |  |  |  |  |  |  |  |  |  |  |  |  |  |  |  |  |  |  |  |  |  |  |  |  |  |
| INT_M2 | .42^***^ | **.59^***^** | .35^***^ | .54^***^ | 1 |  |  |  |  |  |  |  |  |  |  |  |  |  |  |  |  |  |  |  |  |  |  |  |  |  |
| INT_M3 | .29^***^ | .41^***^ | **.51^***^** | .38^***^ | .58^***^ | 1 |  |  |  |  |  |  |  |  |  |  |  |  |  |  |  |  |  |  |  |  |  |  |  |  |
| INT_M4 | .31^***^ | .33^***^ | .42^***^ | .31^***^ | .39^***^ | .61^***^ | 1 |  |  |  |  |  |  |  |  |  |  |  |  |  |  |  |  |  |  |  |  |  |  |  |
| INT_G1 | **.39^***^** | .21^***^ | .15^**^ | **.28^***^** | .14^**^ | .12^**^ | .11^**^ | 1 |  |  |  |  |  |  |  |  |  |  |  |  |  |  |  |  |  |  |  |  |  |  |
| INT_G2 | .35^***^ | **.41^***^** | .18^***^ | .17^***^ | **.38^***^** | .21^***^ | .19^***^ | .51^***^ | 1 |  |  |  |  |  |  |  |  |  |  |  |  |  |  |  |  |  |  |  |  |  |
| INT_G3 | .20^***^ | .24^***^ | **.31^***^** | .10^*^ | .23^***^ | **.41^***^** | .27^***^ | .36^***^ | .52^***^ | 1 |  |  |  |  |  |  |  |  |  |  |  |  |  |  |  |  |  |  |  |  |
| INT_G4 | .21^***^ | .18^***^ | .26^***^ | .13^**^ | .19^***^ | .28^***^ | **.45^***^** | .41^***^ | .42^***^ | .59^***^ | 1 |  |  |  |  |  |  |  |  |  |  |  |  |  |  |  |  |  |  |  |
| INT_E1 | **.34^***^** | .13^***^ | .08^*^ | **.30^***^** | .16^***^ | .09^*^ | .12^**^ | **.47^***^** | .29^***^ | .16^***^ | .25^**^ | 1 |  |  |  |  |  |  |  |  |  |  |  |  |  |  |  |  |  |  |
| INT_E2 | .32^***^ | **.31^***^** | .13^**^ | .21^***^ | **.36^***^** | .17^***^ | .20^***^ | .38^***^ | **.50^***^** | .25^***^ | .26^**^ | .57^***^ | 1 |  |  |  |  |  |  |  |  |  |  |  |  |  |  |  |  |  |
| INT_E3 | .21^***^ | .25^***^ | **.35^***^** | .18^***^ | .29^***^ | **.46^***^** | .33^***^ | .28^***^ | .30^***^ | **.51^***^** | .36^**^ | .39^***^ | .52^***^ | 1 |  |  |  |  |  |  |  |  |  |  |  |  |  |  |  |  |
| INT_E4 | .25^***^ | .24^***^ | .31^***^ | .11^*^ | .18^***^ | .32^***^ | **.46^***^** | .31^***^ | .31^***^ | .37^***^ | **.52^**^** | .32^***^ | .44^***^ | .60^***^ | 1 |  |  |  |  |  |  |  |  |  |  |  |  |  |  |  |
| ACH_M1 | **.17^***^** | .20^***^ | .21^***^ | **.17^***^** | .23^***^ | .15^**^ | .20^***^ | **-.03** | -.10 | -.10 | -.05 | **.00** | .03 | -.01 | .02 | 1 |  |  |  |  |  |  |  |  |  |  |  |  |  |  |
| ACH_M2 | .19^***^ | **.21^***^** | .25^***^ | .18^***^ | **.24^***^** | .19^**^ | .22^***^ | -.07 | **-.12^*^** | -.11 | -.06 | -.09^*^ | **.01** | -.03 | .00 | .64^***^ | 1 |  |  |  |  |  |  |  |  |  |  |  |  |  |
| ACH_M3 | .22^***^ | .19^***^ | **.30^***^** | .28^***^ | .27^***^ | **.24^***^** | .25^***^ | -.05 | -.11 | **-.09** | -.05 | -.01 | .01 | **.04** | -.01 | .66^***^ | .69^***^ | 1 |  |  |  |  |  |  |  |  |  |  |  |  |
| ACH_M4 | .22^***^ | .21^***^ | .35^***^ | .23^***^ | .24^***^ | .22^***^ | **.28^***^** | -.06 | -.10 | -.08 | **-.01** | -.02 | .01 | .08 | **.07** | .63^***^ | .63^***^ | .74^***^ | 1 |  |  |  |  |  |  |  |  |  |  |  |
| ACH_G1 | **.09^**^** | .06 | .06 | **-.03** | .00 | -.03 | -.03 | **-.03** | -.04 | -.12^*^ | -.09 | **-.02** | .02 | -.03 | -.08 | **.37^***^** | .33^***^ | .31^***^ | .29^***^ | 1 |  |  |  |  |  |  |  |  |  |  |
| ACH_G2 | .09^*^ | **.11^**^** | .12^**^ | -.06 | **.03** | -.03 | .00 | -.03 | **-.08** | -.09 | -.05 | -.02 | **.07** | -.04 | -.05 | .41^***^ | **.37^***^** | .36^***^ | .37^***^ | .70^***^ | 1 |  |  |  |  |  |  |  |  |  |
| ACH_G3 | .13^**^ | .12^**^ | **.14^**^** | -.06 | .04 | **.04** | .05 | .00 | .02 | **.01** | -.04 | -.03 | .04 | **-.01** | .00 | .37^***^ | .35^***^ | **.33^***^** | .40^***^ | .58^***^ | .62^***^ | 1 |  |  |  |  |  |  |  |  |
| ACH_G4 | .19^***^ | .18^***^ | .25^***^ | .01 | .07 | .06 | **.11^*^** | .05 | .00 | .00 | **.04** | .01 | .06 | .08 | **.06** | .36^***^ | .36^***^ | .42^***^ | **.47^***^** | .57^***^ | .63^***^ | .62^***^ | 1 |  |  |  |  |  |  |  |
| ACH_E1 | **.09^*^** | .07 | .13^*^ | **-.01** | .02 | -.02 | .00 | **.05** | -.04 | -.03 | -.01 | **.14^**^** | .21^***^ | .12 | .14^**^ | **.37^***^** | .32^***^ | .34^***^ | .29^***^ | **.38^***^** | .39^***^ | .36^***^ | .34^***^ | 1 |  |  |  |  |  |  |
| ACH_E2 | .12^**^ | **.09** | .13^**^ | .04 | **.03** | -.03 | .02 | .08 | **.02** | -.02 | -.03 | .07 | **.19^***^** | .09 | .09^*^ | .38^***^ | **.38^***^** | .38^***^ | .37^***^ | .43^***^ | **.48^***^** | .42^***^ | .44^***^ | .67^***^ | 1 |  |  |  |  |  |
| ACH_E3 | .14^**^ | .14^**^ | **.19^***^** | .04 | .04 | **.01** | .07 | .07 | -.02 | **-.04** | -.06 | .13^*^ | .23^***^ | **.15^*^** | .10^*^ | .33^***^ | .36^***^ | **.39^***^** | .35^***^ | .39^***^ | .44^***^ | **.39^***^** | .44^***^ | .61^***^ | .71^***^ | 1 |  |  |  |  |
| ACH_E4 | .12^*^ | .11^*^ | .15^**^ | -.01 | .02 | -.01 | **.08** | .06 | -.05 | -.09 | **-.02** | .12^*^ | .23^***^ | .17^**^ | **.18^***^** | .35^***^ | .36^***^ | .37^***^ | **.39^***^** | .40^***^ | .46^***^ | .43^***^ | **.51^**^** | .62^***^ | .69^***^ | .75^***^ | 1 |  |  |  |
| Gender^a^ | -.17^***^ | -.13^**^ | -.11* | -.21^***^ | -.16^***^ | -.19^***^ | -.14^**^ | .11^**^ | .07 | .03 | .06 | .04 | .02 | .02 | .04 | -.21^***^ | -.21^***^ | -.15^**^ | -.13^**^ | -.08^**^ | -.05 | .03 | .10^*^ | .09^*^ | .10^**^ | .09^*^ | .08 | 1 |  |  |
| SES | .05 | .01 | .04 | .04 | -.02 | -.04 | .01 | -.09 | -.02 | -.04 | -.09 | -.13^*^ | -.01 | -.09 | -.04 | .12 | .14^*^ | .15^*^ | .18^**^ | .09 | .15^*^ | .16 | .17^*^ | .15 | .16^*^ | .11 | .12 | .03 | 1 |  |
| Class type^b^ | .21^***^ | .25^***^ | .24^***^ | .11^*^ | .13^*^ | .09 | .08 | -.03 | -.08 | -.15 | -.09 | -.02 | .01 | .00 | .00 | .46^***^ | .45^***^ | .44^***^ | .47^***^ | .35^***^ | .41^***^ | .42^***^ | .44^***^ | .33^***^ | .34^***^ | .38^***^ | .39^***^ | -.10^*^ | .31^***^ | 1 |

*Note.* M = mathematics, G = German, E = English, NFC = need for cognition, INT = academic interest, ACH = academic achievement, SES = socioeconomic background. Numbers indicate measurement occasion T1 to T4. Correlations at the same measurement point are shown in bold.

^a^ Gender is coded as 0 = male and 1 = female.

^b^ Class type is coded as 0 = regular class and 1 = gifted class.

* *p* < .05. ** *p* < .01. *** *p* < .001.

Measurement Invariance Testing

Table S4

Results for the Tests of Measurement Invariance over Time for the Need for Cognition and Academic Interest Measures

| Model | χ² | *df* | SCF | *p* | CFI | RMSEA [90% CI] | SRMR | Δχ² (*df*), *p* | ΔCFI | ΔRMSEA | AIC | BIC | adj. BIC |
| --- | --- | --- | --- | --- | --- | --- | --- | --- | --- | --- | --- | --- | --- |
| **Need for Cognition** |  |  |  |  |  |  |  |  |  |  |  |  |  |
| Configural | 945.506 | 459 | 1.169 | <.001 | .959 | .034 [.031, .037] | .033 |  |  |  | 71471.481 | 72122.331 | 71693.588 |
| Metric | 1044.022 | 479 | 1.159 | <.001 | .952 | .036 [.033, .039] | .046 | 112.668 (20), <.001 | .007 | .002 | 71536.883 | 72091.310 | 71726.085 |
| Scalar | 1250.975 | 499 | 1.157 | <.001 | .936 | .041 [.038, .043] | .054 | 214.008 (20), <.001 | .016 | .005 | 71734.294 | 72192.299 | 71890.591 |
| Partial scalar^a^ | 1141.768 | 493 | 1.158 | <.001 | .945 | .038 [.035, .041] | .051 | 99.793 (14), <.001 | .007 | .002 | 71620.250 | 72107.181 | 71786.418 |
| **Academic Interest** |  |  |  |  |  |  |  |  |  |  |  |  |  |
| Mathematics |  |  |  |  |  |  |  |  |  |  |  |  |  |
| Configural | 49.787 | 30 | 1.039 | .013 | .992 | .027 [.012, .040] | .023 |  |  |  | 25525.362 | 25814.759 | 25624.206 |
| Metric | 53.728 | 36 | 1.029 | .029 | .993 | .023 [.008, .035] | .028 | 3.634 (6), .073 | .001 | .004 | 25516.894 | 25777.352 | 25605.854 |
| Scalar | 65.957 | 42 | 1.002 | .011 | .990 | .025 [.012, .036] | .030 | 12.860 (6), .045 | .003 | .002 | 25515.734 | 25747.252 | 25594.809 |
| German |  |  |  |  |  |  |  |  |  |  |  |  |  |
| Configural | 60.778 | 30 | 1.221 | .001 | .994 | .033 [.021, .045] | .029 |  |  |  | 24420.487 | 24709.885 | 24519.332 |
| Metric | 70.103 | 36 | 1.171 | .001 | .993 | .032 [.021, .043] | .032 | 8.557 (6), .200 | .001 | .001 | 24416.382 | 24676.839 | 24505.342 |
| Scalar | 95.144 | 42 | 1.167 | <.001 | .990 | .037 [.027, .047] | .033 | 25.321 (6), <.001 | .003 | .005 | 24433.288 | 24664.806 | 24512.364 |
| English |  |  |  |  |  |  |  |  |  |  |  |  |  |
| Configural | 46.865 | 30 | 1.272 | .026 | .996 | .025 [.009, .038] | .021 |  |  |  | 22456.467 | 22745.864 | 22555.311 |
| Metric | 55.637 | 36 | 1.313 | .019 | .995 | .024 [.010, .036] | .030 | 8.853 (6), .182 | .001 | .001 | 22457.875 | 22718.333 | 22546.835 |
| Scalar | 66.745 | 42 | 1.274 | .009 | .994 | .025 [.013, .036] | .033 | 11.521 (6), .074 | .001 | .001 | 22457.865 | 22689.383 | 22536.940 |

Note. SCF = scaling correction factor; CFI = comparative fit index; RMSEA = root-mean-square error of approximation; SRMR = standardized root mean square residual; AIC = Akaike information criterion; BIC = Bayesian information criterion; adj. BIC = adjusted Bayesian information criterion.

^a^ Intercepts of items 1, 3, and 9 were freely estimated.

Results for the Autoregressive Cross-Lagged Panel Models

Table S5

Parameters for Model 1 and Model 2 (Achievement Added) by Domain

| Parameter |  | Mathematics |  |  |  | German |  |  |  | English |  |
| --- | --- | --- | --- | --- | --- | --- | --- | --- | --- | --- | --- |
|  | Stand. estimates | 95% CI | *p* |  | Stand. estimates | 95% CI | *p* |  | Stand. estimates | 95% CI | *p* |
| **Model 1** |  |  |  |  |  |  |  |  |  |  |  |
| Regression Weights |  |  |  |  |  |  |  |  |  |  |  |
| NFC2 on |  |  |  |  |  |  |  |  |  |  |  |
| NFC1 | 0.620 | [0.557, 0.683] | < .001 |  | 0.647 | [0.586, 0.708] | < .001 |  | 0.666 | [0.611, 0.721] | < .001 |
| INT1 | 0.021 | [-0.044, 0.086] | .536 |  | -0.041 | [-0.112, 0.030] | .259 |  | -0.094 | [-0.153, -0.035] | .002 |
| NFC3 on |  |  |  |  |  |  |  |  |  |  |  |
| NFC2 | 0.526 | [0.416, 0.636] | < .001 |  | 0.572 | [0.464, 0.680] | < .001 |  | 0.568 | [0.482, 0.654] | < .001 |
| INT2 | 0.051 | [-0.045, 0.147] | .291 |  | -0.040 | [-0.144, 0.064] | .443 |  | -0.042 | [-0.124, 0.040] | .319 |
| INT2 on |  |  |  |  |  |  |  |  |  |  |  |
| NFC1 | 0.168 | [0.076, 0.260] | < .001 |  | 0.169 | [0.095, 0.243] | < .001 |  | 0.141 | [0.072, 0.210] | < .001 |
| INT1 | 0.449 | [0.373, 0.525] | < .001 |  | 0.453 | [0.384, 0.522] | < .001 |  | 0.524 | [0.465, 0.583] | < .001 |
| INT3 on |  |  |  |  |  |  |  |  |  |  |  |
| NFC2 | 0.101 | [0.023, 0.179] | .012 |  | 0.031 | [-0.071, 0.133] | .549 |  | 0.095 | [0.005, 0.185] | .040 |
| INT2 | 0.529 | [0.453, 0.605] | < .001 |  | 0.523 | [0.419, 0.627] | < .001 |  | 0.510 | [0.416, 0.604] | < .001 |
| INT4 on |  |  |  |  |  |  |  |  |  |  |  |
| NFC3 | 0.155 | [0.069, 0.241] | < .001 |  | 0.078 | [-0.008, 0.164] | .072 |  | 0.111 | [0.003, 0.219] | .045 |
| INT3 | 0.531 | [0.441, 0.621] | < .001 |  | 0.575 | [0.510, 0.640] | < .001 |  | 0.572 | [0.486, 0.658] | < .001 |
| Correlations |  |  |  |  |  |  |  |  |  |  |  |
| NFC1 with INT1 | 0.567 | [0.502, 0.632] | < .001 |  | 0.393 | [0.336, 0.450] | < .001 |  | 0.344 | [0.273, 0.415] | < .001 |
| NFC2 with INT2 | 0.498 | [0.406, 0.590] | < .001 |  | 0.313 | [0.233, 0.393] | < .001 |  | 0.246 | [0.152, 0.340] | < .001 |
| NFC3 with INT3 | 0.394 | [0.296, 0.492] | < .001 |  | 0.297 | [0.179, 0.415] | < .001 |  | 0.339 | [0.223, 0.455] | < .001 |
| **Model 2** |  |  |  |  |  |  |  |  |  |  |  |
| Regression Weights |  |  |  |  |  |  |  |  |  |  |  |
| NFC2 on |  |  |  |  |  |  |  |  |  |  |  |
| NFC1 | 0.609 | [0.544, 0.674] | < .001 |  | 0.649 | [0.586, 0.712] | < .001 |  | 0.664 | [0.607, 0.721] | < .001 |
| INT1 | 0.011 | [-0.056, 0.078] | .736 |  | -0.041 | [-0.112, 0.030] | .253 |  | -0.097 | [-0.156, -0.038] | .001 |
| ACH1 | 0.087 | [0.014, 0.160] | .020 |  | -0.005 | [-0.046, 0.036] | .798 |  | 0.030 | [-0.058, 0.118] | .504 |
| NFC3 on |  |  |  |  |  |  |  |  |  |  |  |
| NFC2 | 0.511 | [0.403, 0.619] | < .001 |  | 0.563 | [0.457, 0.669] | < .001 |  | 0.567 | [0.483, 0.651] | < .001 |
| INT2 | 0.018 | [-0.080, 0.116] | .713 |  | -0.031 | [-0.131, 0.069] | .539 |  | -0.066 | [-0.152, 0.020] | .134 |
| ACH2 | 0.150 | [0.074, 0.226] | < .001 |  | 0.064 | [-0.020, 0.148] | .139 |  | 0.110 | [0.020, 0.200] | .015 |
| INT2 on |  |  |  |  |  |  |  |  |  |  |  |
| NFC1 | 0.151 | [0.059, 0.243] | .001 |  | 0.177 | [0.103, 0.251] | < .001 |  | 0.135 | [0.068, 0.202] | < .001 |
| INT1 | 0.440 | [0.366, 0.514] | < .001 |  | 0.448 | [0.377, 0.519] | < .001 |  | 0.510 | [0.451, 0.569] | < .001 |
| ACH1 | 0.130 | [0.050, 0.210] | .002 |  | -0.053 | [-0.129, 0.023] | .174 |  | 0.140 | [0.066, 0.214] | < .001 |
| INT3 on |  |  |  |  |  |  |  |  |  |  |  |
| NFC2 | 0.098 | [0.022, 0.174] | .014 |  | 0.042 | [-0.056, 0.140] | .401 |  | 0.094 | [0.004, 0.184] | .041 |
| INT2 | 0.519 | [0.433, 0.605] | < .001 |  | 0.513 | [0.411, 0.615] | < .001 |  | 0.503 | [0.407, 0.599] | < .001 |
| ACH2 | 0.042 | [-0.056, 0.140] | .405 |  | -0.056 | [-0.130, 0.018] | .141 |  | 0.030 | [-0.058, 0.118] | .509 |
| INT4 on |  |  |  |  |  |  |  |  |  |  |  |
| NFC3 | 0.140 | [0.052, 0.228] | .002 |  | 0.087 | [0.005, 0.169] | .040 |  | 0.108 | [-0.004, 0.220] | .059 |
| INT3 | 0.518 | [0.420, 0.616] | < .001 |  | 0.571 | [0.506, 0.636] | < .001 |  | 0.568 | [0.478, 0.658] | < .001 |
| ACH3 | 0.077 | [-0.019, 0.173] | .115 |  | -0.048 | [-0.130, 0.034] | .250 |  | 0.026 | [-0.048, 0.100] | .489 |
| ACH2 on |  |  |  |  |  |  |  |  |  |  |  |
| NFC1 | 0.056 | [-0.009, 0.121] | .088 |  | 0.031 | [-0.024, 0.086] | .272 |  | 0.077 | [-0.017, 0.171] | .106 |
| INT1 | 0.055 | [-0.012, 0.122] | .105 |  | -0.023 | [-0.068, 0.022] | .322 |  | -0.041 | [-0.149, 0.067] | .454 |
| ACH1 | 0.627 | [0.558, 0.696] | < .001 |  | 0.700 | [0.641, 0.759] | < .001 |  | 0.669 | [0.591, 0.747] | < .001 |
| ACH3 on |  |  |  |  |  |  |  |  |  |  |  |
| NFC2 | -0.015 | [-0.107, 0.077] | .751 |  | 0.032 | [-0.052, 0.116] | .456 |  | 0.049 | [-0.037, 0.135] | .270 |
| INT2 | 0.120 | [0.018, 0.222] | .022 |  | 0.053 | [-0.041, 0.147] | .275 |  | 0.081 | [0.008, 0.154] | .027 |
| ACH2 | 0.668 | [0.599, 0.737] | < .001 |  | 0.632 | [0.567, 0.697] | < .001 |  | 0.686 | [0.633, 0.739] | < .001 |
| ACH4 on |  |  |  |  |  |  |  |  |  |  |  |
| NFC3 | 0.166 | [0.076, 0.256] | < .001 |  | 0.192 | [0.125, 0.259] | < .001 |  | -0.019 | [-0.084, 0.046] | .575 |
| INT3 | -0.039 | [-0.106, 0.028] | .251 |  | -0.069 | [-0.143, 0.005] | .065 |  | 0.069 | [-0.013, 0.151] | .103 |
| ACH3 | 0.698 | [0.631, 0.765] | < .001 |  | 0.603 | [0.538, 0.668] | < .001 |  | 0.745 | [0.702, 0.788] | < .001 |
| Correlations |  |  |  |  |  |  |  |  |  |  |  |
| NFC1 with |  |  |  |  |  |  |  |  |  |  |  |
| INT1 | 0.567 | [0.502, 0.632] | < .001 |  | 0.393 | [0.336, 0.450] | < .001 |  | 0.344 | [0.273, 0.415] | < .001 |
| ACH1 | 0.172 | [0.103, 0.241] | < .001 |  | 0.097 | [0.032, 0.162] | .003 |  | 0.078 | [-0.014, 0.170] | .095 |
| NFC2 with |  |  |  |  |  |  |  |  |  |  |  |
| INT2 | 0.491 | [0.403, 0.579] | < .001 |  | 0.313 | [0.233, 0.393] | < .001 |  | 0.242 | [0.150, 0.334] | < .001 |
| ACH2 | 0.049 | [-0.033, 0.131] | .248 |  | 0.099 | [0.019, 0.179] | .017 |  | -0.028 | [-0.120, 0.064] | .545 |
| NFC3 with |  |  |  |  |  |  |  |  |  |  |  |
| INT3 | 0.388 | [0.292, 0.484] | < .001 |  | 0.305 | [0.187, 0.423] | < .001 |  | 0.338 | [0.226, 0.450] | < .001 |
| ACH3 | 0.155 | [0.059, 0.251] | .002 |  | 0.061 | [-0.041, 0.163] | .245 |  | 0.114 | [0.036, 0.192] | .005 |
| INT1 with ACH1 | 0.166 | [0.082, 0.250] | < .001 |  | -0.026 | [-0.097, 0.045] | .469 |  | 0.124 | [0.034, 0.214] | .007 |
| INT2 with ACH2 | 0.064 | [-0.016, 0.144] | .122 |  | -0.077 | [-0.171, 0.017] | .110 |  | 0.108 | [0.012, 0.204] | .029 |
| INT3 with ACH3 | 0.095 | [0.017, 0.173] | .017 |  | 0.041 | [-0.059, 0.141] | .417 |  | 0.057 | [-0.057, 0.171] | .325 |
| INT4 with ACH4 | 0.105 | [0.001, 0.209] | .048 |  | 0.072 | [-0.016, 0.160] | .109 |  | 0.143 | [0.033, 0.253] | .011 |

*Note.* NFC = Need for Cognition; INT = academic interest; ACH = academic achievement; SES = socioeconomic status.

Table S6

Model Fit Indices for Model 1 and Model 2 With Control Variables by Domain

| Model | χ^2^ | *df* | *p* | SCF | CFI | RMSEA [90% CI] | SRMR |
| --- | --- | --- | --- | --- | --- | --- | --- |
| Model 1 |  |  |  |  |  |  |  |
| Mathematics | 2012.425 | 1041 | < .001 | 1.115 | .944 | .032 [.030, .034] | .046 |
| German | 1991.634 | 1041 | < .001 | 1.141 | .948 | .031 [.029, .034] | .053 |
| English | 1992.911 | 1041 | < .001 | 1.154 | .947 | .031 [.029, .034] | .049 |
| Model 2 |  |  |  |  |  |  |  |
| Mathematics | 2399.800 | 1206 | < .001 | 1.096 | .939 | .033 [.031, .035] | .047 |
| German | 2409.336 | 1206 | < .001 | 1.122 | .940 | .033 [.031, .035] | .053 |
| English | 2301.970 | 1206 | < .001 | 1.125 | .945 | .031 [.029, .033] | .048 |

Note. SCF = scaling correction factor; CFI = comparative fit index; RMSEA = root mean square error of approximation; SRMR = standardized root mean square residual.

Figure S1

*Results of the Autoregressive Cross-Lagged Panel Models 1 and 2 Including Control Variables Gender, SES, and Class Type*

*Note*. Standardized coefficients in mathematics, German, and English are presented for the models controlling for gender, SES, and class type. For path coefficients of the control variables, see Table S7. Structural arrows are bold when coefficients are significant in at least two domains. NFC = need for cognition; INT = interest in mathematics, German, or English; ACH = academic achievement in mathematics, German, or English.

^✝^ *p* < .100. ^*^ *p* < .050. ^**^ *p* < .010. ^***^ *p* < .001.

Table S7

Parameters for Model 1 and Model 2 With Control Variables by Domain

| Parameter |  | Mathematics |  |  |  | German |  |  |  | English |  |
| --- | --- | --- | --- | --- | --- | --- | --- | --- | --- | --- | --- |
|  | Stand. estimates | 95% CI | *p* |  | Stand. estimates | 95% CI | *p* |  | Stand. estimates | 95% CI | *p* |
| **Model 1** |  |  |  |  |  |  |  |  |  |  |  |
| Regression Weights |  |  |  |  |  |  |  |  |  |  |  |
| NFC1 on |  |  |  |  |  |  |  |  |  |  |  |
| Gender | -0.145 | [-0.212, -0.078] | < .001 |  | -0.144 | [-0.211, -0.077] | < .001 |  | -0.147 | [-0.214, -0.080] | < .001 |
| SES | -0.034 | [-0.183, 0.115] | .657 |  | -0.040 | [-0.177, 0.097] | .566 |  | -0.028 | [-0.169, 0.113] | .700 |
| Class type | 0.204 | [0.106, 0.302] | < .001 |  | 0.206 | [0.110, 0.302] | < .001 |  | 0.202 | [0.106, 0.298] | < .001 |
| NFC2 on |  |  |  |  |  |  |  |  |  |  |  |
| NFC1 | 0.588 | [0.519, 0.657] | < .001 |  | 0.614 | [0.547, 0.681] | < .001 |  | 0.638 | [0.577, 0.699] | < .001 |
| INT1 | 0.023 | [-0.044, 0.090] | .495 |  | -0.029 | [-0.107, 0.049] | .472 |  | -0.091 | [-0.150, -0.032] | .003 |
| Gender | -0.015 | [-0.076, 0.046] | .627 |  | -0.012 | [-0.073, 0.049] | .698 |  | -0.007 | [-0.066, 0.052] | .810 |
| SES | -0.073 | [-0.214, 0.068] | .314 |  | -0.054 | [-0.197, 0.089] | .456 |  | -0.062 | [-0.195, 0.071] | .359 |
| Class type | 0.146 | [0.056, 0.236] | .002 |  | 0.135 | [0.047, 0.223] | .002 |  | 0.133 | [0.043, 0.223] | .004 |
| NFC3 on |  |  |  |  |  |  |  |  |  |  |  |
| NFC2 | 0.492 | [0.378, 0.606] | < .001 |  | 0.531 | [0.423, 0.639] | < .001 |  | 0.533 | [0.447, 0.619] | < .001 |
| INT2 | 0.053 | [-0.043, 0.149] | .282 |  | -0.015 | [-0.117, 0.087] | .767 |  | -0.032 | [-0.114, 0.050] | .446 |
| Gender | -0.024 | [-0.093, 0.045] | .491 |  | -0.023 | [-0.086, 0.040] | .484 |  | -0.028 | [-0.091, 0.035] | .375 |
| SES | -0.009 | [-0.168, 0.150] | .910 |  | -0.003 | [-0.146, 0.140] | .965 |  | -0.013 | [-0.162, 0.136] | .860 |
| Class type | 0.118 | [0.026, 0.210] | .012 |  | 0.110 | [0.022, 0.198] | .016 |  | 0.115 | [0.027, 0.203] | .011 |
| INT1 on |  |  |  |  |  |  |  |  |  |  |  |
| Gender | -0.199 | [-0.272, -0.126] | < .001 |  | 0.117 | [0.041, 0.193] | .002 |  | 0.042 | [-0.023, 0.107] | .206 |
| SES | 0.015 | [-0.110, 0.140] | .813 |  | -0.129 | [-0.278, 0.020] | .089 |  | -0.153 | [-0.286, -0.020] | .025 |
| Class type | 0.080 | [-0.012, 0.172] | .087 |  | 0.023 | [-0.087, 0.133] | .682 |  | 0.033 | [-0.075, 0.141] | .545 |
| INT2 on |  |  |  |  |  |  |  |  |  |  |  |
| NFC1 | 0.151 | [0.051, 0.251] | .003 |  | 0.210 | [0.136, 0.284] | < .001 |  | 0.150 | [0.081, 0.219] | < .001 |
| INT1 | 0.447 | [0.373, 0.521] | < .001 |  | 0.436 | [0.362, 0.510] | < .001 |  | 0.528 | [0.465, 0.591] | < .001 |
| Gender | -0.034 | [-0.107, 0.039] | .351 |  | 0.045 | [-0.028, 0.118] | .226 |  | 0.026 | [-0.035, 0.087] | .404 |
| SES | -0.078 | [-0.198, 0.042] | .203 |  | 0.067 | [-0.041, 0.175] | .217 |  | 0.063 | [-0.051, 0.177] | .284 |
| Class type | 0.074 | [-0.028, 0.176] | .151 |  | -0.130 | [-0.261, 0.001] | .052 |  | -0.028 | [-0.124, 0.068] | .571 |
| INT3 on |  |  |  |  |  |  |  |  |  |  |  |
| NFC2 | 0.100 | [0.024, 0.176] | .010 |  | 0.070 | [-0.032, 0.172] | .180 |  | 0.098 | [-0.004, 0.200] | .057 |
| INT2 | 0.516 | [0.434, 0.598] | < .001 |  | 0.498 | [0.398, 0.598] | < .001 |  | 0.505 | [0.409, 0.601] | < .001 |
| Gender | -0.097 | [-0.177, -0.017] | .019 |  | -0.006 | [-0.082, 0.070] | .877 |  | 0.023 | [-0.063, 0.109] | .596 |
| SES | 0.018 | [-0.133, 0.169] | .814 |  | -0.005 | [-0.144, 0.134] | .943 |  | -0.096 | [-0.261, 0.069] | .253 |
| Class type | -0.015 | [-0.121, 0.091] | .777 |  | -0.122 | [-0.238, -0.006] | .038 |  | 0.007 | [-0.113, 0.127] | .907 |
| INT4 on |  |  |  |  |  |  |  |  |  |  |  |
| NFC3 | 0.158 | [0.072, 0.244] | < .001 |  | 0.089 | [-0.011, 0.189] | .083 |  | 0.126 | [0.016, 0.236] | .025 |
| INT3 | 0.526 | [0.430, 0.622] | < .001 |  | 0.566 | [0.486, 0.646] | < .001 |  | 0.569 | [0.487, 0.651] | < .001 |
| Gender | -0.032 | [-0.120, 0.056] | .473 |  | 0.045 | [-0.039, 0.129] | .296 |  | 0.037 | [-0.026, 0.100] | .248 |
| SES | 0.035 | [-0.069, 0.139] | .512 |  | -0.080 | [-0.209, 0.049] | .223 |  | 0.020 | [-0.129, 0.169] | .788 |
| Class type | -0.023 | [-0.145, 0.099] | .712 |  | 0.005 | [-0.118, 0.128] | .933 |  | -0.038 | [-0.128, 0.052] | .407 |
| Correlations |  |  |  |  |  |  |  |  |  |  |  |
| NFC1 with INT1 | 0.548 | [0.477, 0.619] | < .001 |  | 0.430 | [0.371, 0.489] | < .001 |  | 0.365 | [0.298, 0.432] | < .001 |
| NFC2 with INT2 | 0.491 | [0.395, 0.587] | < .001 |  | 0.346 | [0.275, 0.417] | < .001 |  | 0.256 | [0.158, 0.354] | < .001 |
| NFC3 with INT3 | 0.398 | [0.302, 0.494] | < .001 |  | 0.319 | [0.209, 0.429] | < .001 |  | 0.345 | [0.233, 0.457] | < .001 |
| Gender with |  |  |  |  |  |  |  |  |  |  |  |
| SES | 0.029 | [-0.073, 0.131] | .585 |  | 0.029 | [-0.075, 0.133] | .585 |  | 0.026 | [-0.082, 0.134] | .640 |
| Class type | -0.095 | [-0.177, -0.013] | .024 |  | -0.095 | [-0.177, -0.013] | .024 |  | -0.095 | [-0.177, -0.013] | .024 |
| SES with class type | 0.317 | [0.197, 0.437] | < .001 |  | 0.314 | [0.196, 0.432] | < .001 |  | 0.306 | [0.186, 0.426] | < .001 |
| **Model 2** |  |  |  |  |  |  |  |  |  |  |  |
| Regression Weights |  |  |  |  |  |  |  |  |  |  |  |
| NFC1 on |  |  |  |  |  |  |  |  |  |  |  |
| Gender | -0.144 | [-0.213, -0.075] | < .001 |  | -0.144 | [-0.211, -0.077] | < .001 |  | -0.147 | [-0.214, -0.080] | < .001 |
| SES | -0.043 | [-0.319, 0.233] | .761 |  | -0.035 | [-0.188, 0.118] | .654 |  | -0.022 | [-0.169, 0.125] | .773 |
| Class type | 0.206 | [0.094, 0.318] | < .001 |  | 0.204 | [0.106, 0.302] | < .001 |  | 0.200 | [0.104, 0.296] | < .001 |
| NFC2 on |  |  |  |  |  |  |  |  |  |  |  |
| NFC1 | 0.588 | [0.519, 0.657] | < .001 |  | 0.616 | [0.547, 0.685] | < .001 |  | 0.638 | [0.577, 0.699] | < .001 |
| INT1 | 0.017 | [-0.052, 0.086] | .617 |  | -0.030 | [-0.110, 0.050] | .458 |  | -0.092 | [-0.155, -0.029] | .004 |
| ACH1 | 0.033 | [-0.053, 0.119] | .442 |  | -0.052 | [-0.099, -0.005] | .032 |  | 0.004 | [-0.086, 0.094] | .931 |
| Gender | -0.012 | [-0.083, 0.059] | .737 |  | -0.014 | [-0.077, 0.049] | .667 |  | -0.007 | [-0.064, 0.050] | .806 |
| SES | -0.058 | [-0.399, 0.283] | .738 |  | -0.055 | [-0.200, 0.090] | .460 |  | -0.062 | [-0.195, 0.071] | .360 |
| Class type | 0.127 | [-0.004, 0.258] | .060 |  | 0.154 | [0.062, 0.246] | .001 |  | 0.131 | [0.039, 0.223] | .005 |
| NFC3 on |  |  |  |  |  |  |  |  |  |  |  |
| NFC2 | 0.494 | [0.378, 0.610] | < .001 |  | 0.531 | [0.425, 0.637] | < .001 |  | 0.536 | [0.452, 0.620] | < .001 |
| INT2 | 0.027 | [-0.071, 0.125] | .591 |  | -0.015 | [-0.115, 0.085] | .771 |  | -0.050 | [-0.136, 0.036] | .250 |
| ACH2 | 0.117 | [0.039, 0.195] | .004 |  | 0.025 | [-0.057, 0.107] | .545 |  | 0.077 | [-0.017, 0.171] | .113 |
| Gender | -0.012 | [-0.085, 0.061] | .744 |  | -0.021 | [-0.086, 0.044] | .518 |  | -0.038 | [-0.101, 0.025] | .239 |
| SES | -0.017 | [-0.264, 0.230] | .894 |  | -0.010 | [-0.159, 0.139] | .891 |  | -0.018 | [-0.165, 0.129] | .805 |
| Class type | 0.073 | [-0.049, 0.195] | .238 |  | 0.103 | [0.005, 0.201] | .039 |  | 0.090 | [-0.004, 0.184] | .065 |
| INT1 on |  |  |  |  |  |  |  |  |  |  |  |
| Gender | -0.198 | [-0.276, -0.120] | < .001 |  | 0.117 | [0.039, 0.195] | .003 |  | 0.042 | [-0.023, 0.107] | .197 |
| SES | 0.000 | [-0.288, 0.288] | 1.000 |  | -0.126 | [-0.291, 0.039] | .137 |  | -0.152 | [-0.283, -0.021] | .024 |
| Class type | 0.085 | [-0.035, 0.205] | .163 |  | 0.022 | [-0.094, 0.138] | .704 |  | 0.034 | [-0.074, 0.142] | .543 |
| INT2 on |  |  |  |  |  |  |  |  |  |  |  |
| NFC1 | 0.148 | [0.052, 0.244] | .003 |  | 0.211 | [0.137, 0.285] | < .001 |  | 0.150 | [0.083, 0.217] | < .001 |
| INT1 | 0.438 | [0.367, 0.509] | < .001 |  | 0.434 | [0.361, 0.507] | < .001 |  | 0.507 | [0.438, 0.576] | < .001 |
| ACH1 | 0.133 | [0.059, 0.207] | < .001 |  | -0.015 | [-0.076, 0.046] | .644 |  | 0.155 | [0.075, 0.235] | < .001 |
| Gender | -0.014 | [-0.090, 0.062] | .717 |  | 0.044 | [-0.029, 0.117] | .225 |  | 0.009 | [-0.054, 0.072] | .782 |
| SES | -0.063 | [-0.308, 0.182] | .613 |  | 0.063 | [-0.049, 0.175] | .267 |  | 0.064 | [-0.052, 0.180] | .276 |
| Class type | 0.011 | [-0.107, 0.129] | .859 |  | -0.124 | [-0.255, 0.007] | .065 |  | -0.080 | [-0.174, 0.014] | .093 |
| INT3 on |  |  |  |  |  |  |  |  |  |  |  |
| NFC2 | 0.103 | [0.025, 0.181] | .011 |  | 0.072 | [-0.030, 0.174] | .163 |  | 0.099 | [-0.003, 0.201] | .056 |
| INT2 | 0.505 | [0.417, 0.593] | < .001 |  | 0.495 | [0.395, 0.595] | < .001 |  | 0.499 | [0.403, 0.595] | < .001 |
| ACH2 | 0.036 | [-0.062, 0.134] | .469 |  | -0.011 | [-0.084, 0.062] | .762 |  | 0.029 | [-0.069, 0.127] | .568 |
| Gender | -0.093 | [-0.177, -0.009] | .030 |  | -0.006 | [-0.082, 0.070] | .869 |  | 0.020 | [-0.068, 0.108] | .654 |
| SES | 0.011 | [-0.150, 0.172] | .888 |  | 0.005 | [-0.134, 0.144] | .945 |  | -0.106 | [-0.271, 0.059] | .209 |
| Class type | -0.027 | [-0.133, 0.079] | .615 |  | -0.122 | [-0.240, -0.004] | .041 |  | 0.000 | [-0.118, 0.118] | .999 |
| INT4 on |  |  |  |  |  |  |  |  |  |  |  |
| NFC3 | 0.149 | [0.059, 0.239] | .001 |  | 0.091 | [-0.009, 0.191] | .074 |  | 0.125 | [0.013, 0.237] | .029 |
| INT3 | 0.511 | [0.415, 0.607] | < .001 |  | 0.569 | [0.487, 0.651] | < .001 |  | 0.566 | [0.480, 0.652] | < .001 |
| ACH3 | 0.096 | [-0.004, 0.196] | .059 |  | -0.051 | [-0.143, 0.041] | .276 |  | 0.024 | [-0.060, 0.108] | .583 |
| Gender | -0.023 | [-0.111, 0.065] | .616 |  | 0.047 | [-0.035, 0.129] | .271 |  | 0.034 | [-0.033, 0.101] | .317 |
| SES | 0.035 | [-0.073, 0.143] | .526 |  | -0.078 | [-0.223, 0.067] | .295 |  | 0.025 | [-0.130, 0.180] | .749 |
| Class type | -0.061 | [-0.188, 0.066] | .348 |  | 0.026 | [-0.111, 0.163] | .713 |  | -0.047 | [-0.151, 0.057] | .381 |
| ACH1 on |  |  |  |  |  |  |  |  |  |  |  |
| Gender | -0.163 | [-0.247, -0.079] | < .001 |  | -0.048 | [-0.099, 0.003] | .060 |  | 0.115 | [0.042, 0.188] | .002 |
| SES | -0.052 | [-0.583, 0.479] | .848 |  | 0.003 | [-0.244, 0.250] | .982 |  | 0.007 | [-0.201, 0.215] | .949 |
| Class type | 0.458 | [0.284, 0.632] | < .001 |  | 0.350 | [0.219, 0.481] | < .001 |  | 0.341 | [0.208, 0.474] | < .001 |
| ACH2 on |  |  |  |  |  |  |  |  |  |  |  |
| NFC1 | 0.025 | [-0.038, 0.088] | .444 |  | -0.005 | [-0.072, 0.062] | .890 |  | 0.065 | [-0.025, 0.155] | .156 |
| INT1 | 0.058 | [-0.001, 0.117] | .053 |  | -0.002 | [-0.053, 0.049] | .953 |  | -0.025 | [-0.123, 0.073] | .614 |
| ACH1 | 0.533 | [0.451, 0.615] | < .001 |  | 0.641 | [0.584, 0.698] | < .001 |  | 0.622 | [0.540, 0.704] | < .001 |
| Gender | -0.064 | [-0.137, 0.009] | .085 |  | 0.016 | [-0.047, 0.079] | .617 |  | 0.062 | [0.009, 0.115] | .021 |
| SES | 0.042 | [-0.234, 0.318] | .767 |  | 0.047 | [-0.088, 0.182] | .492 |  | 0.017 | [-0.097, 0.131] | .767 |
| Class type | 0.173 | [0.008, 0.338] | .038 |  | 0.165 | [0.073, 0.257] | < .001 |  | 0.126 | [-0.017, 0.269] | .082 |
| ACH3 on |  |  |  |  |  |  |  |  |  |  |  |
| NFC2 | -0.060 | [-0.150, 0.030] | .192 |  | -0.022 | [-0.104, 0.060] | .606 |  | 0.013 | [-0.069, 0.095] | .759 |
| INT2 | 0.143 | [0.045, 0.241] | .004 |  | 0.082 | [-0.008, 0.172] | .072 |  | 0.101 | [0.030, 0.172] | .005 |
| ACH2 | 0.595 | [0.532, 0.658] | < .001 |  | 0.554 | [0.476, 0.632] | < .001 |  | 0.632 | [0.569, 0.695] | < .001 |
| Gender | 0.004 | [-0.072, 0.080] | .917 |  | 0.064 | [-0.016, 0.144] | .121 |  | 0.031 | [-0.028, 0.090] | .312 |
| SES | -0.031 | [-0.617, 0.555] | .917 |  | -0.024 | [-0.271, 0.223] | .851 |  | -0.045 | [-0.161, 0.071] | .451 |
| Class type | 0.183 | [-0.029, 0.395] | .090 |  | 0.224 | [0.085, 0.363] | .002 |  | 0.173 | [0.067, 0.279] | .001 |
| ACH4 on |  |  |  |  |  |  |  |  |  |  |  |
| NFC3 | 0.129 | [0.043, 0.215] | .004 |  | 0.161 | [0.090, 0.232] | < .001 |  | -0.053 | [-0.122, 0.016] | .129 |
| INT3 | -0.022 | [-0.093, 0.049] | .554 |  | -0.036 | [-0.110, 0.038] | .351 |  | 0.086 | [-0.002, 0.174] | .054 |
| ACH3 | 0.628 | [0.559, 0.697] | < .001 |  | 0.528 | [0.448, 0.608] | < .001 |  | 0.698 | [0.639, 0.757] | < .001 |
| Gender | -0.015 | [-0.080, 0.050] | .643 |  | 0.122 | [0.057, 0.187] | < .001 |  | 0.012 | [-0.057, 0.081] | .743 |
| SES | 0.031 | [-0.079, 0.141] | .578 |  | 0.041 | [-0.139, 0.221] | .655 |  | 0.002 | [-0.125, 0.129] | .978 |
| Class type | 0.158 | [0.070, 0.246] | < .001 |  | 0.167 | [0.065, 0.269] | .001 |  | 0.144 | [0.034, 0.254] | .011 |
| Correlations |  |  |  |  |  |  |  |  |  |  |  |
| NFC1 with |  |  |  |  |  |  |  |  |  |  |  |
| INT1 | 0.548 | [0.477, 0.619] | < .001 |  | 0.430 | [0.371, 0.489] | < .001 |  | 0.366 | [0.299, 0.433] | < .001 |
| ACH1 | 0.058 | [-0.020, 0.136] | .146 |  | 0.018 | [-0.043, 0.079] | .553 |  | 0.043 | [-0.047, 0.133] | .358 |
| NFC2 with |  |  |  |  |  |  |  |  |  |  |  |
| INT2 | 0.493 | [0.395, 0.591] | < .001 |  | 0.345 | [0.272, 0.418] | < .001 |  | 0.259 | [0.163, 0.355] | < .001 |
| ACH2 | 0.027 | [-0.049, 0.103] | .488 |  | 0.070 | [-0.018, 0.158] | .117 |  | -0.041 | [-0.133, 0.051] | .386 |
| NFC3 with |  |  |  |  |  |  |  |  |  |  |  |
| INT3 | 0.392 | [0.298, 0.486] | < .001 |  | 0.321 | [0.211, 0.431] | < .001 |  | 0.345 | [0.237, 0.453] | < .001 |
| ACH3 | 0.145 | [0.049, 0.241] | .003 |  | 0.035 | [-0.063, 0.133] | .478 |  | 0.091 | [0.015, 0.167] | .018 |
| INT1 with ACH1 | 0.100 | [0.018, 0.182] | .017 |  | -0.011 | [-0.093, 0.071] | .796 |  | 0.153 | [0.069, 0.237] | < .001 |
| INT2 with ACH2 | 0.070 | [-0.012, 0.152] | .091 |  | -0.061 | [-0.141, 0.019] | .137 |  | 0.111 | [0.021, 0.201] | .015 |
| INT3 with ACH3 | 0.104 | [0.031, 0.177] | .004 |  | 0.074 | [-0.028, 0.176] | .155 |  | 0.049 | [-0.065, 0.163] | .394 |
| INT4 with ACH4 | 0.126 | [0.022, 0.230] | .017 |  | 0.073 | [-0.029, 0.175] | .162 |  | 0.154 | [0.044, 0.264] | .006 |
| Gender with |  |  |  |  |  |  |  |  |  |  |  |
| SES | 0.024 | [-0.096, 0.144] | .693 |  | 0.029 | [-0.083, 0.141] | .618 |  | 0.031 | [-0.075, 0.137] | .568 |
| Class type | -0.095 | [-0.177, -0.013] | .024 |  | -0.095 | [-0.177, -0.013] | .024 |  | -0.095 | [-0.177, -0.013] | .024 |
| SES with class type | 0.314 | [0.183, 0.445] | < .001 |  | 0.318 | [0.200, 0.436] | < .001 |  | 0.306 | [0.188, 0.424] | < .001 |

Note. Gender is coded as 0 = male and 1 = female. Class type is coded as 0 = regular class and 1 = gifted class. NFC = Need for Cognition; INT = academic interest; ACH = academic achievement; SES = socioeconomic status.

Analysis Code: Mplus Syntax for Model 2 With Control Variables Included

variable:

usevariables are

nfc1_1 nfc2_1 nfc3_1 nfc5_1 nfc12_1 nfc13_1 nfc16_1 nfc17_1 nfc18_1 nfc19_1 nfc20_1 nfc1_2 nfc2_2 nfc3_2 nfc5_2 nfc12_2 nfc13_2 nfc16_2 nfc17_2 nfc18_2 nfc19_2 nfc20_2 nfc1_3 nfc2_3 nfc3_3 nfc5_3 nfc12_3 nfc13_3 nfc16_3 nfc17_3 nfc18_3 nfc19_3 nfc20_3 xmoint11 xmoint21 xmoint31 xmoint12 xmoint22 xmoint32 xmoint13 xmoint23 xmoint33 xmoint14 xmoint24 xmoint34 ACH_X1 ACH_X2 ACH_X3 ACH_X4 gender ses class type;

cluster = class;

analysis:

type = complex;

estimator = MLR;

starts = 20;

model:

!need for cognition: positive items

nfc1 by nfc1_1*(L1) nfc2_1(L2) nfc3_1(L3) nfc5_1(L4) nfc12_1(L5) nfc13_1(L6) nfc16_1(L7) nfc17_1(L8) nfc18_1(L9) nfc19_1(L10) nfc20_1(L11);

nfc2 by nfc1_2*(L1) nfc2_2(L2) nfc3_2(L3) nfc5_2(L4) nfc12_2(L5) nfc13_2(L6) nfc16_2(L7) nfc17_2(L8) nfc18_2(L9) nfc19_2(L10) nfc20_2(L11);

nfc3 by nfc1_3*(L1) nfc2_3(L2) nfc3_3(L3) nfc5_3(L4) nfc12_3(L5) nfc13_3(L6) nfc16_3(L7) nfc17_3(L8) nfc18_3(L9) nfc19_3(L10) nfc20_3(L11);

!correlations between the same items over time

nfc1_1 with nfc1_2 nfc1_3;

nfc2_1 with nfc2_2 nfc2_3;

nfc3_1 with nfc3_2 nfc3_3;

nfc5_1 with nfc5_2 nfc5_3;

nfc12_1 with nfc12_2 nfc12_3;

nfc13_1 with nfc13_2 nfc13_3;

nfc16_1 with nfc16_2 nfc16_3;

nfc17_1 with nfc17_2 nfc17_3;

nfc18_1 with nfc18_2 nfc18_3;

nfc19_1 with nfc19_2 nfc19_3;

nfc20_1 with nfc20_2 nfc20_3;

nfc1_2 with nfc1_3;

nfc2_2 with nfc2_3;

nfc3_2 with nfc3_3;

nfc5_2 with nfc5_3;

nfc12_2 with nfc12_3;

nfc13_2 with nfc13_3;

nfc16_2 with nfc16_3;

nfc17_2 with nfc17_3;

nfc18_2 with nfc18_3;

nfc19_2 with nfc19_3;

nfc20_2 with nfc20_3;

[nfc1 - nfc3*]

[nfc1_1](i1);

[nfc2_1](i2);

[nfc3_1](i3);

[nfc5_1](i4);

[nfc12_1](i5);

[nfc13_1](i6);

[nfc16_1](i7);

[nfc17_1](i8);

[nfc18_1](i9);

[nfc19_1](i10);

[nfc20_1](i11);

[nfc1_2];

[nfc2_2](i2);

[nfc3_2];

[nfc5_2](i4);

[nfc12_2](i5);

[nfc13_2](i6);

[nfc16_2](i7);

[nfc17_2](i8);

[nfc18_2];

[nfc19_2](i10);

[nfc20_2](i11);

[nfc1_3];

[nfc2_3](i2);

[nfc3_3];

[nfc5_3](i4);

[nfc12_3](i5);

[nfc13_3](i6);

[nfc16_3](i7);

[nfc17_3](i8);

[nfc18_3];

[nfc19_3](i10);

[nfc20_3](i11);

!domain-specific interest

xmoint1 by xmoint11*(LLL1) xmoint21(LLL2) xmoint31(LLL3);

xmoint2 by xmoint12*(LLL1) xmoint22(LLL2) xmoint32(LLL3);

xmoint3 by xmoint13*(LLL1) xmoint23(LLL2) xmoint33(LLL3);

xmoint4 by xmoint14*(LLL1) xmoint24(LLL2) xmoint34(LLL3);

[xmoint1 - xmoint4*]

[xmoint11](iii1);

[xmoint21](iii2);

[xmoint31](iii3);

[xmoint12](iii1);

[xmoint22](iii2);

[xmoint32](iii3);

[xmoint13](iii1);

[xmoint23](iii2);

[xmoint33](iii3);

[xmoint14](iii1);

[xmoint24](iii2);

[xmoint34](iii3);

!correlations between the same items over time

xmoint11 with xmoint12 xmoint13 xmoint14;

xmoint21 with xmoint22 xmoint23 xmoint24;

xmoint31 with xmoint32 xmoint33 xmoint34;

xmoint12 with xmoint13 xmoint14;

xmoint22 with xmoint23 xmoint24;

xmoint32 with xmoint33 xmoint34;

xmoint13 with xmoint14;

xmoint23 with xmoint24;

xmoint33 with xmoint34;

!cross-lagged panel with control variables

nfc1 on gender ses class type;

nfc2 on nfc1 ACH_X1 xmoint1 gender ses class type;

nfc3 on nfc2 ACH_X2 xmoint2 gender ses class type;

xmoint1 on gender ses class type;

xmoint2 on nfc1 ACH_X1 xmoint1 gender ses class type;

xmoint3 on nfc2 ACH_X2 xmoint2 gender ses class type;

xmoint4 on nfc3 ACH_X3 xmoint3 gender ses class type;

ACH_X1 on gender ses class type;

ACH_X2 on nfc1 ACH_X1 xmoint1 gender ses class type;

ACH_X3 on nfc2 ACH_X2 xmoint2 gender ses class type;

ACH_X4 on nfc3 ACH_X3 xmoint3 gender ses class type;

!(residual) correlations

nfc1 with ACH_X1 xmoint1;

nfc2 with ACH_X2 xmoint2;

nfc3 with ACH_X3 xmoint3;

ACH_X1 with xmoint1;

ACH_X2 with xmoint2;

ACH_X3 with xmoint3;

ACH_X4 with xmoint4;

gender with ses class type;

ses with class type;

!effect coding

model constraint:

L1 = 11 - L2 - L3 - L4 - L5 - L6 - L7 - L8 - L9 - L10 - L11;

i2 = 0 - i1 - i3 -i4 -i5 -i6 -i7 -i8 -i9 -i10 -i11;

LLL1 = 3- LLL2 - LLL3;

iii1 = 0 - iii2 - iii3;

References

Bächtold, M., Papet, J., Asensio, D. B., Borne, S., Checchi, K. de, Jeziorski, A., Gabriel, P., & Cassignol, F. (2023). Effects of motivation, evaluativism, and perceived social support on deep approach to learning at university. *European Journal of Psychology of Education.* Advance online publication. <https://doi.org/10.1007/s10212-023-00722-4>

Bergold, S., & Steinmayr, R. (2023). The interplay between investment traits and cognitive abilities: Investigating reciprocal effects in elementary school age. *Child Development.* Advance online publication. <https://doi.org/10.1111/cdev.14029>

Bråten, I., Anmarkrud, Ø., Brandmo, C., & Strømsø, H. I. (2014). Developing and testing a model of direct and indirect relationships between individual differences, processing, and multiple-text comprehension. *Learning and Instruction*, *30*, 9–24. <https://doi.org/10.1016/j.learninstruc.2013.11.002>

Cazan, A.‑M., & Indreica, S. E. (2014). Need for cognition and approaches to learning among university students. *Procedia - Social and Behavioral Sciences*, *127*, 134–138. <https://doi.org/10.1016/j.sbspro.2014.03.227>

Day, E. A., Espejo, J., Kowollik, V., Boatman, P. R., & McEntire, L. E. (2007). Modeling the links between need for cognition and the acquisition of a complex skill. *Personality and Individual Differences*, *42*, 201–212. <https://doi.org/10.1016/j.paid.2006.06.012>

Dickhäuser, O., & Reinhard, M.‑A. (2010). How students build their performance expectancies: The importance of need for cognition. *European Journal of Psychology of Education*, *25*, 399–409. <https://doi.org/10.1007/s10212-010-0027-4>

Embretson, S. E., & Reise, S. P. (2000). *Item response theory for psychologists* (1^st^ ed.). Psychology Press. <https://doi.org/10.4324/9781410605269>

Evans, C. J., Kirby, J. R., & Fabrigar, L. R. (2003). Approaches to learning, need for cognition, and strategic flexibility among university students. *The British Journal of Educational Psychology*, *73*, 507–528. <https://doi.org/10.1348/000709903322591217>

Gogol, K., Brunner, M., Martin, R., Preckel, F., & Goetz, T. (2017). Affect and motivation within and between school subjects: Development and validation of an integrative structural model of academic self-concept, interest, and anxiety. *Contemporary Educational Psychology*, *49*, 46–65. <https://doi.org/10.1016/j.cedpsych.2016.11.003>

Keller, U., Strobel, A., Wollschläger, R., Greiff, S., Martin, R., Vainikainen, M.‑P., & Preckel, F. (2016). A need for cognition scale for children and adolescents. *European Journal of Psychological Assessment*, *35*, 137–149. <https://doi.org/10.1027/1015-5759/a000370>

Kolen, M. J., & Brennan, R. L. (2014). *Test equating, scaling, and linking: Methods and practices* (3^rd^ ed.). *Statistics for Social and Behavioral Sciences*. Springer. <https://doi.org/10.1007/978-1-4939-0317-7>

Kramer, A.‑W., van Duijvenvoorde, A. C., Krabbendam, L., & Huizenga, H. M. (2021). Individual differences in adolescents’ willingness to invest cognitive effort: Relation to need for cognition, motivation and cognitive capacity. *Cognitive Development*, *57*, 100978. <https://doi.org/10.1016/j.cogdev.2020.100978>

Luong, C., Strobel, A., Wollschläger, R., Greiff, S., Vainikainen, M.‑P., & Preckel, F. (2017). Need for cognition in children and adolescents: Behavioral correlates and relations to academic achievement and potential. *Learning and Individual Differences*, *53*, 103–113. <https://doi.org/10.1016/j.lindif.2016.10.019>

Olson, K., Camp, C., & Fuller, D. (1984). Curiosity and Need for Cognition. *Psychological Reports*, *54*, 71–74. <https://doi.org/10.2466/pr0.1984.54.1.71>

Preckel, F. (2014). Assessing need for cognition in early adolescence. *European Journal of Psychological Assessment*, *30*, 65–72. <https://doi.org/10.1027/1015-5759/a000170>

Robitzsch, A., Kiefer, T., & Wu, M. L. (2019). *TAM: Test analysis modules*. R package version 3.1-45. <https://CRAN.R-project.org/package=TAM>

Scherrer, V., Preckel, F., Schmidt, I., & Elliot, A. J. (2020). Development of achievement goals and their relation to academic interest and achievement in adolescence: A review of the literature and two longitudinal studies. *Developmental Psychology*, *56*(4), 795–814. <https://doi.org/10.1037/dev0000898>

Soemer, A., & Schiefele, U. (2019). Text difficulty, topic interest, and mind wandering during reading. *Learning and Instruction*, *61*, 12–22. <https://doi.org/10.1016/j.learninstruc.2018.12.006>

Tang, X., Renninger, K. A., Hidi, S. E., Murayama, K., Lavonen, J., & Salmela‐Aro, K. (2022). The differences and similarities between curiosity and interest: Meta-analysis and network analyses. *Learning and Instruction*, *80*, 101628. <https://doi.org/10.1016/j.learninstruc.2022.101628>

Trautwein, U., Lüdtke, O., Nagy, N., Lenski, A., Niggli, A., & Schnyder, I. (2015). Using individual interest and conscientiousness to predict academic effort: Additive, synergistic, or compensatory effects? *Journal of Personality and Social Psychology*, *109*, 142–162. <https://doi.org/10.1037/pspp0000034>

Warm, T. A. (1989). Weighted likelihood estimation of ability in item response theory. *Psychometrika*, *54*, 427–450. <https://doi.org/10.1007/BF02294627>

Wu, M. L., Adams, R. J., Wilson, M. R., & Haldane, S. A. (2007). *ACER ConQuest: Generalised item response modelling software* (Version 2.0) [Computer software]. <https://www.acer.org/au/conquest>
